# DNA barcoding the fishes of Lizard Island (Great Barrier Reef)

**DOI:** 10.3897/BDJ.5.e12409

**Published:** 2017-04-13

**Authors:** Dirk Steinke, Jeremy R deWaard, Martin F Gomon, Jeffrey W Johnson, Helen K Larson, Oliver Lucanus, Glenn I Moore, Sally Reader, Robert D Ward

**Affiliations:** 1 Centre for Biodiversity Genomics - University of Guelph, Guelph, Canada; 2 Department of Integrative Biology - University of Guelph, Guelph, Canada; 3 Museum Victoria, Melbourne, Australia; 4 Queensland Museum, PO Box 3300, South Brisbane QLD 4101, Australia, Brisbane, Australia; 5 Museum and Art Gallery of the Northern Territory, Darwin, Australia; 6 Belowwater, Montreal, Canada; 7 Western Australian Museum, Perth, Australia; 8 Australian Museum, Sidney, Australia; 9 CSIRO Marine and Atmospheric Research, Hobart, Australia

**Keywords:** DNA barcoding, taxonomy, coral reef, biodiversity

## Introduction

DNA barcoding is a relatively new and powerful species identification tool that has found ready application to many animal taxa. It is based on sequencing a 650 bp region of the mitochondrial cytochrome c oxidase gene I (COI), the premise being that different species have different COI sequences or barcodes (Hebert et al. 2003). A reference library of verified barcodes is constructed that can be queried against to determine the identity of sequences derived from unknown specimens and sources.

There have been several large-scale projects aimed at verifying its potential for discriminating fish (Hubert et al. 2008, Steinke et al. 2009, Steinke et al. 2016a, Ward et al. 2005), and the international FISH-BOL campaign (Ward et al. 2009, Becker et al. 2011) has as its ultimate goal the provision of reference barcodes for all the world’s fish species. There are more than 30,000 fish species, and collecting and sequencing them all is an enormous task. As of January 2017, FISH-BOL had barcoded approximately 14,400 species (from 221,500 specimens), so there is still a long way to go to attain its ultimate goal.

One strategy that can be used to increase species coverage is a barcoding ‘blitz’ of a chosen region (Telfer et al. 2015). This is essentially a short but intensive effort to collect and barcode as many species as possible from that region.

**Figure 1.**
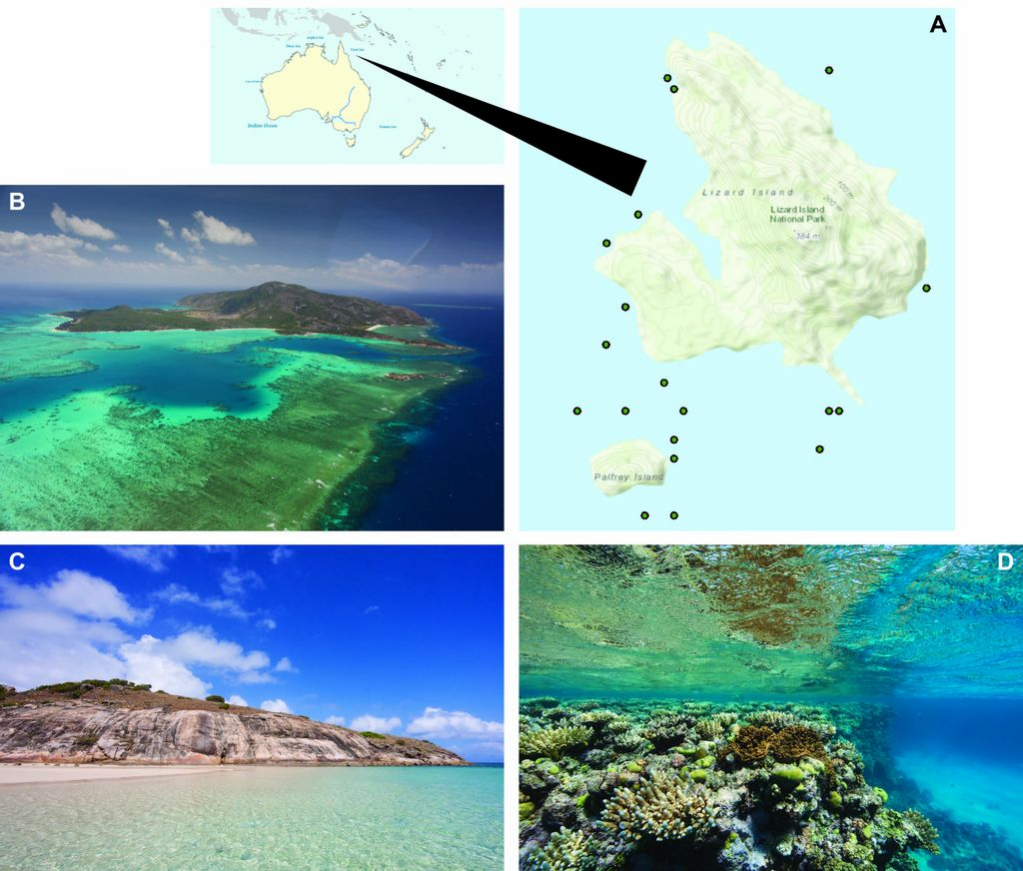
Lizard Island Group. A. Map of Lizard Island with collection sites for specimens examined in this study. B. Aerial photo view from the South. C. Typical coastal features of the island group. D. Typical reef edge near Lizard Island. (Photo credits Oliver Lucanus)

The Australian fish fauna is remarkably rich with many endemic species. Some 4600 species have been estimated for Australian waters, about 2300 of which have been recorded from the Great Barrier Reef (Hoese et al. 2015). Lizard Island is a small island (~1000 hectares) located in the northern section of the Great Barrier Reef (Fig. 1), and is home to an extraordinary diversity of fishes (~1500 species), from cryptic reef dwelling to fast swimming open water species (Hutchings et al. 2008). Importantly, it is also home to the Lizard Island Research Station. This facility, owned and operated by the Australian Museum and supported by the Lizard Island Reef Research Foundation, provides good accommodation and laboratory facilities, including a number of small motor boats.

In September 2008, a team of 12 Australian and Canadian scientists (see Acknowledgments) spent two weeks on the island, collecting, photographing and tissue sampling as many fish species as could be caught, under permit conditions provided by the Great Barrier Reef Marine Park Authority (GBRMPA). DNA sequencing was subsequently done at the Centre for Biodiversity Genomics in Guelph, Canada. We describe here the results of this barcoding blitz on the fishes of Lizard Island.

## Materials and Methods

### Sampling

Members of the team arrived on Lizard Island on 3 September 2008 and departed 17-19 September. Fish were collected at different sites around Lizard Island under GBRMPA permit G26633, using clove oil, handnets, spears, light traps, beach seines, and hook and line approaches. Reefs, bommies, beaches, and mangroves were visited (Fig. 1).

Prior to processing the specimens were morphologically identified by the appropriate expert using available taxonomic keys, field guides, and distribution records. Each specimen was assigned to one of five levels of reliability depending on the taxonomic expertise of the identifier involved and their intentions, following guidelines developed by Commonwealth Scientific and Industrial Research Organisation (CSIRO) fish taxonomists, and laid out in the FISH-BOL sampling protocol (Steinke and Hanner 2011). A general definition of these levels follows:

Level 1: highly reliable identification - specimen identified by an recognized authority of the group, or a specialist that is presently studying or has reviewed the group in the region in question.

Level 2: identification made with high degree of confidence at all levels - specimen identified by a trained identifier who had prior knowledge of the group in the region or used available literature to identify the specimen.

Level 3: identification made with high confidence to genus but less so to species - specimen identified by a trained identifier who was confident of its generic placement but did not substantiate their species identification using the literature, or a trained identifier who used the literature, but still could not make a positive identification to species, or an untrained identifier who used most of the available literature to make the identification.

Level 4: identification made with limited confidence - specimen identified by a trained identifier who was confident of its family placement, but unsure of generic or species identifications (no literature used apart from illustrations), or an untrained identifier who had/used limited literature to make the identification.

Level 5: identification superficial - specimen identified by a trained identifier who is uncertain ofthe family placement of the species (cataloging identification only), an untrained identifier using, at best, figures in a guide, or where the status and expertise of the identifier is unknown.

For this study, we collected 1,075 individuals, which were found to represent 395 fish species, from the waters around Lizard Island. When possible, several adults were analyzed per species. Specimens were kept on ice and subsequently imaged in the field (by convention left side of the animal). Samples used for DNA analysis were removed of lateral muscle from the right side of the specimen or by removing the right eye from very small specimens such as juveniles. Specimens are stored as vouchers in the Australian Museum, Sydney, the Australian National Fish Collection at CSIRO, Hobart, and the Western Australian Museum, Perth. Collection details are recorded in the public dataset DS-LIFE (http://dx.doi.org/10.5883/DS-LIFE) on the Barcode of Life Data Systems database (BOLD, http://www.boldsystems.org, see Ratnasingham and Hebert 2007).

### DNA extraction, amplification and sequencing

DNA was extracted from the muscle tissue of each specimen using an automated glass Fiber (AcroPrep) method (Ivanova et al. 2006). The 650 bp barcode region of COI was subsequently amplified under the following thermal conditions: 2 min at 95°C; 35 cycles of 0.5 min at 94°C, 0.5 min at 52°C, and 1 min at 72°C; 10 min at 72°C; then held at 4°C. The 12.5 µl PCR reaction mixes included 6.25 µl of 10% trehalose, 2.00 µl of ultrapure water, 1.25 µl 10X PCR buffer [200 mM Tris-HCl (pH 8.4), 500 mM KCl], 0.625 µl MgCl_2_ (50 mM), 0.125 µl of each primer cocktail (0.01 mM, using primer cocktails C_FishF1t1 and C_FishR1t1 or C_VF1LFt1 and C_VR1LRt1 (Ivanova et al. 2007), 0.062 µl of each dNTP (10 mM), 0.060 µl of Platinum® Taq Polymerase (Invitrogen), and 2.0 µl of DNA template. PCR amplicons were visualized on a 1.2% agarose gel E-Gel® (Invitrogen) and bidirectionally sequenced using sequencing primers M13F or M13R and the BigDye® Terminator v.3.1 Cycle Sequencing Kit (Applied Biosystems, Inc.) on an ABI 3730xl capillary sequencer following manufacturer's instructions (for more detail and alternatives see Steinke et al. 2016b). Bi-directional sequences were assembled and edited using either SEQSCAPE v.2.1.1 (Applied Biosystems) or CodonCode Aligner software (CodonCode Corporation, USA) prior to their upload to BOLD.

### Sequence Analysis

We used the analysis tools in BOLD to calculate the nucleotide composition of the sequences and distributions of Kimura-2-Parameter distances (Kimura 1980) within and between species. Relationships among individuals and species were visualized with a NJ tree based on K2P distances (Suppl. material 3). In addition, all barcodes were assigned a Barcode Index Number (BIN) as implemented in BOLD (Ratnasingham and Hebert 2013). BIN assignments on BOLD are constantly updated as new sequences are added, and individual BINs can be split or merged as new data are obtained (Ratnasingham and Hebert 2013). BIN assignments were used for data curation, interpretation of species boundaries, and to flag potential cryptic species.

A list of all marine fish species (N=2764) currently present in Queensland was obtained from the Australian Faunal Directory in December 2016 (Hoese et al. 2015). It was used to determine geographical barcode coverage and to identify potential new records found by this study. A digital version of this checklist (CL-QUFIS - Queensland fishes) is publically available on BOLD (http://dx.doi.org/10.5883/CL-QUFIS).

Sequence data are available on both BOLD and GenBank. Specimen and collection data, sequences, specimen images, GenBank accession numbers, and trace files can be found in the public dataset DS-LIFE on BOLD (http://dx.doi.org/10.5883/DS-LIFE). An abbreviated version of the data is available in Suppl. material 1.

## Results

**Figure 2.**
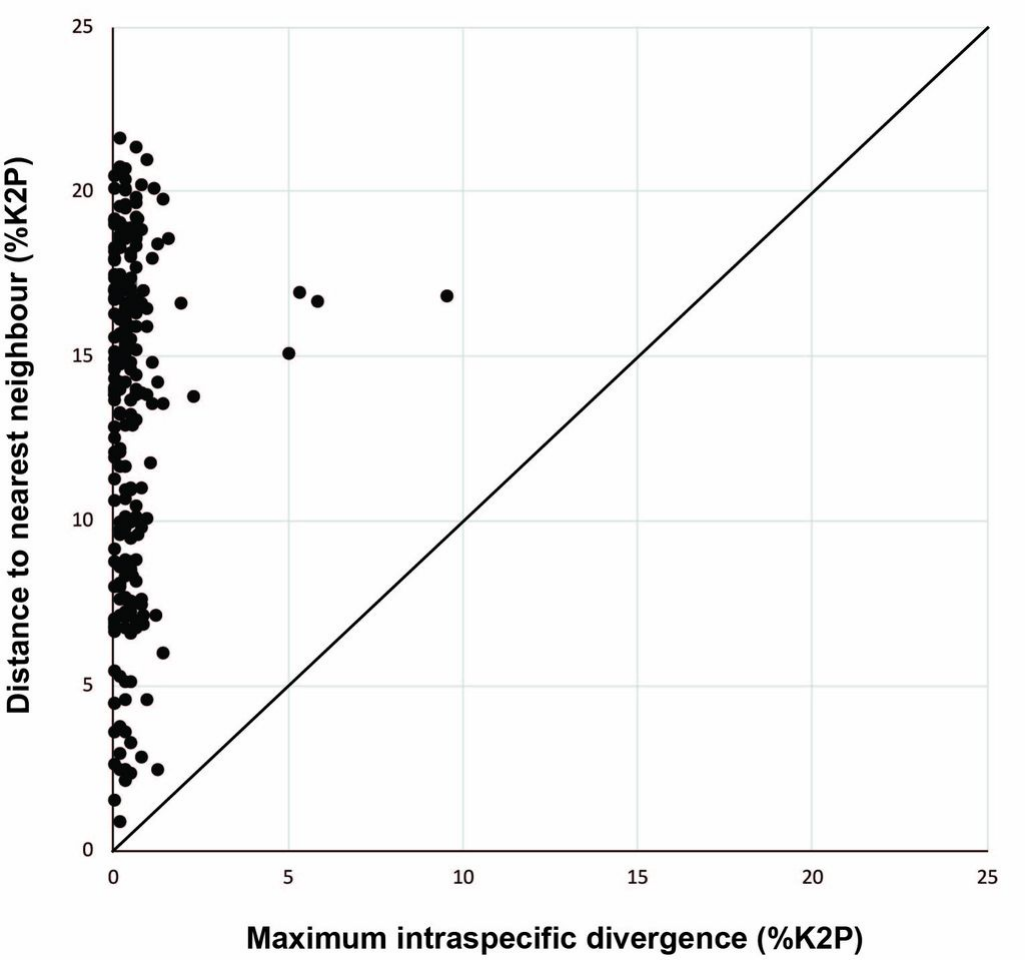
Plot of COI maximum intraspecific divergence versus mean nearest-neighbour distance for 235 species of marine fishes from Lizard Island represented by two or more individuals. Points above the diagonal line indicate species with a barcode gap.

For this study, we obtained 983 sequence records that derived from 358 named species (177 genera, 59 families) and another 13 species that could only be reliably identified to genus level (Table 1, Suppl. material 2). An additional 24 species failed to provide any useful sequences (Suppl. material 2). Among the successfully sequenced species, 235 were represented by two or more individuals. They possessed intraspecific divergences averaging 0.3% (with a mean of the maximum intraspecific divergences of 0.49%), while the mean distance to the Nearest Neighbour taxon was 43-fold higher, averaging 12.96%. As a consequence, there was a clear barcode gap for all species (Fig. 2, Suppl. material 3). Overall nucleotide frequencies were C (28.7%), T (29.3%), A (23.4%), G (18.6%).

All sequences met the quality (<1% N) and length (>500 bp) criteria for BIN assignment, and were assigned to 375 BINs. There was perfect correspondence between the specimens assigned to a particular BIN and the members of a particular morphospecies in nearly all cases (372 of 375). The three exceptions each involved a BIN split with the members of a particular species assigned to two BINs (Table 2).

**Figure 3.**
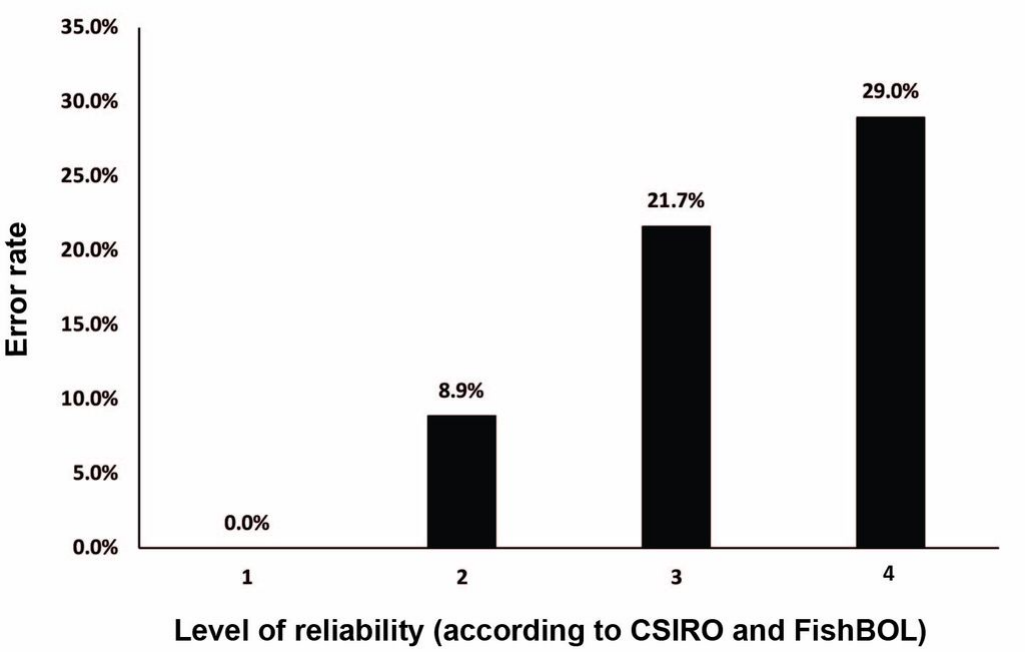
Identification error rates by level of reliability depending on the taxonomic expertise of the identifier involved (in concordance with guidelines of CSIRO and FishBOL).

828 (84%) of the 983 barcoded specimens were correctly identified in the field by one of the fish taxonomists in the team (MG, JJ, PL, GM, SR, AH, see acknowledgements), 106 (11%) were initially misidentified, and 59 (6%) could not be identified to species level, receiving either a genus or family designation. Misidentifications were later exposed and resolved after DNA barcoding analysis and morphological re-inspection by experts with particular taxonomic knowledge. Identification errors in the field occurred more frequently when the identifier indicated a lower level of confidence, reflecting varying degrees of expertise (Fig. 3).

The species detected in the present study were compared to the list of all fishes (N =2764) known from the marine waters of Queensland (Hoese et al. 2015). Only one species we found, *Nectamia similis*, Fraser, 2008, was hitherto unknown from Queensland waters. The 13 insufficiently identified or provisional species were excluded from this analysis, as there could be no occurrence data for these. Overall, the 2008 Lizard Island expedition provided DNA barcodes for 13% of all marine fish species known to occur in Queensland. An analysis utilizing BOLD’s checklist function reveals about 78% of this fauna has now been barcoded following this blitz.

## Discussion

This study assembled DNA barcode sequences for 371 species of marine fishes that occur in the waters of Lizard Island (Table S1). This represents about 25% of the known marine ichthyofauna of the region (Hutchings et al. 2008) and included one species (*Nectamia similis*) that was previously not recorded for Lizard Island nor the entire Great Barrier Reef. These records are the result of a single biotic survey conducted over a two-week period.

Our study also revealed three cases of BIN splits involving the following taxa:

1. *Amniataba caudavittata* (Richardson 1845)

The five specimens of Yellowtail trumpeter *A. caudavittata* fell into two BINs (n=2, n=3) that show 5% sequence divergence between BINs but with novariation within BINs. This genus contains only three described species and one of those (*A. perco)* is barcode divergent (c. 7%) from both BINs. The other species (*A. affinis*) is known only from river systems and lagoons of Papua New Guinea and has not been barcoded. It seems likely that, despite no obvious morphological diversity, the two BINs comprise the original *A. caudavittata* and an overlooked cryptic species that requires description.

2. *Ellochelon vaigiensis* (Quoy and Gaimard 1825)

Specimens of *E. vaigiensis* were represented by two quite divergent (4.9%) BINs (n=5, n=1), which might reflect an instance of unjustified synonymization as several species (*Mugil macrolepidotus, M. melanochir, M. tegobuan, M. occidentalis, M. ventricosus*) have been recently synonymized under this species name (Kottelat 2013). Comparisons with additional publicly available data on BOLD indicate four different lineages under the name E. *vaigensis*. Although further sampling as well as genetic and morphological analysis of type material is required, we suggest that the two lineages detected at Lizard Island may represent distinct species.

3. *Gobiodon quinquestrigatus* (Valenciennes 1837)

Although COI divergence was quite low (1.24%), *G. quinquestrigatus* sequences were placed in two BINs (n=5, n=1). We were not able to find any morphological differences between members of these BINs nor any prior history of names-in-waiting. Without further evidence (e.g.,additional nuclear markers), this instance might either represent the discovery of a cryptic species or an artifact of the BIN algorithm due to high intraspecifc sequence variability and low sampling intensity. Valid species can harbour multiple mtDNA lineages with no morphological differences. With increased sampling such lineages can dissolve.

The speed of conducting this inventory reflected the team’s focus on a single group of organisms and the variety of collecting protocols deployed. One disadvantage of this type of fieldwork is the lack of time and resources available for proper initial identification of the difficult-to-delineate taxa. We identified samples in the field to one of five levels of reliability, depending on the taxonomic expertise of the identifier involved, following a standard protocol of the CSIRO Australia (see Materials and Methods, and Steinke and Hanner 2011). Subsequent to the expedition, we actively pursued expert determinations based on the collected vouchers, photographs, andthe barcode results to refine the taxonomy of the samples. In total, about 11% of the initial field identifications were found to be incorrect, with errors mostly at the level of species. Generally, the identifier was aware when there was a higher risk of initial misidentification because we found that a lower self-identified level of confidence correlates with an increased error rate (Fig. 3). It should be noted that all field participants involved in the species identification were experienced Australian fish researchers, some of whom were experts in particular taxonomic groups. In these cases, identifications were done with the highest level of confidence and such identifications were always show to be correct.

Although the project started as a rapid and intensive effort to collect and barcode as many species as possible from Lizard Island, it took several years to validate and confirm the species IDs. Some inventoried taxa still lack species-level determination, but these will be resolved over time. The barcodes obtained during this study, in concert with the BIN system of BOLD, facilitate crowd sourcing of the necessary taxonomic refinement (e.g. Johnson and Worthington Wilmer 2015). For example, if two specimens are collected in unrelated projects and locations, but assigned to the same BIN, any taxonomic determination on one specimen will bevisible to all researchers involved as the pertinent data are shared on the public BIN page.

Lizard Island is a unique natural reserve with the infrastructure necessary to conduct research on the northern section of the Great Barrier Reef, a barcode reference library and updated species inventory for its fishes adds to the infrastructure that can be shared with present and future researchers. This database is likely to become of increasing significance. In April 2014, Cyclone Ita passed directly across theisland – the most severe storm ever recorded for this location. The storms caused massive coral loss, further amplified by higher than average water temperatures in 2015 and 2016, which led to massive coral bleaching. The latter affected mostly the northern Great Barrier Reef, and one of the worst hit areas was around Lizard Island where about 90% of the coral died (Australian Research Council (ARC) Centre of Excellence for Coral Reef Studies 2016). Such a dramatic environmental change will have a profound impact on the local ichthyofauna, and the observations of our study will become part of a baseline (among studies such as Hoese et al. 2015, Hutchings et al. 2008) allowing us to assess long-term impacts of the bleaching event and better understand how the system might recover.

## Supplementary Material

Supplementary material 1 Summary data for the 983 fish specimens successfully barcoded as part of the Lizard Island barcode 'blitz'. Data type: Table
File: oo_123816.xlsx Steinke, D, deWaard, JR, Gomon, MF, Johnson, JW, Larson, HK, Lucanus, O, Moore, GI, Reader, S, Ward, RD

Supplementary material 2 Species collected at Lizard Island listed by order and family. An asterisk indicates species for which a barcode sequence could not be obtained. Data type: Table
File: oo_123821.docx Steinke, D, deWaard, JR, Gomon, MF, Johnson, JW, Larson, HK, Lucanus, O, Moore, GI, Reader, S, Ward, RD

Supplementary material 3 Neighbour Joining tree based on K2P distances Data type: PDF
File: oo_123831.pdf Steinke, D, deWaard, JR, Gomon, MF, Johnson, JW, Larson, HK, Lucanus, O, Moore, GI, Reader, S, Ward, RD

## Figures and Tables

**Table 1.** Species that could not be identified below genus level.

**Species name**	**Order/Family**	**BIN**	**N**
Cheilodipterus cf. quinquelineatus	Kurtiformes/Apogonidae	BOLD:AAC7857	1
*Eviota* sp.	Gobiiformes/Gobiidae	BOLD:AAB8856	1
*Eviota* sp. 1	Gobiiformes/Gobiidae	BOLD:AAW8200	1
*Eviota* sp. 2	Gobiiformes/Gobiidae	BOLD:AAD2732	4
*Eviota* sp. 3	Gobiiformes/Gobiidae	BOLD:AAD2731	1
*Eviota* sp. 5	Gobiiformes/Gobiidae	BOLD:AAY4519	1
*Gobiodon* sp.	Gobiiformes/Gobiidae	BOLD:AAD0248	1
*Paragobiodon* sp.	Gobiiformes/Gobiidae	BOLD:AAD0247	1
*Salarias* sp.	Blenniiformes/Blenniidae	BOLD:AAB7190	1
*Scarus* sp.	Labriformes/Scaridae	BOLD:ADB4663	1
*Scorpaena* sp.	Scorpaeniformes/Scropeanidae	BOLD:AAE9847	1
*Trimma* oki group 8	Gobiiformes/Gobiidae	BOLD:AAB3909	1
*Trimmatom* sp.	Gobiiformes/Gobiidae	BOLD:AAY4517	2

**Table 2.** Discordances between BIN and species assignments (Species assigned to two BINs)

**Species name**	**Order/Family**	**BIN 1**	**BIN 2**
*Amniataba caudavittata*	Perciformes/Terapontidae	BOLD:AAE2733	BOLD:AAE2734
*Ellochelon vaigiensis*	Mugiliformes/Mugilidae	BOLD:ACK7668	BOLD:AAC9398
*Gobiodon quinquestrigatus*	Gobiiformes/Gobiidae	BOLD:ACF5842	BOLD:AAB5279
