# Supplementary material for: DNA barcoding the fishes of Lizard Island (Great Barrier Reef)
Source: Biodivers Data J. 2017 Apr 13;(5):e12409. doi: 10.3897/BDJ.5.e12409 (PMC5515069; doi:10.3897/BDJ.5.e12409)
Supplement: Supplementary material 2 — Species collected at Lizard Island listed by order and family. An asterisk indicates species for which a barcode sequence could not be obtained. [file bdj-05-e12409-s002.docx]

**Table S2:** Species collected at Lizard Island listed by order and family. An asterisk indicates species for which a barcode sequence could not be obtained.

| Order | Family | *Species* |
| --- | --- | --- |
| Acanthuriformes | Acanthuridae | *Acanthurus blochii* |
| Acanthuriformes | Acanthuridae | *Acanthurus lineatus* |
| Acanthuriformes | Acanthuridae | *Acanthurus nigricauda* |
| Acanthuriformes | Acanthuridae | *Acanthurus nigrofuscus* |
| Acanthuriformes | Acanthuridae | *Acanthurus olivaceus* |
| Acanthuriformes | Acanthuridae | *Acanthurus pyroferus* |
| Acanthuriformes | Acanthuridae | *Ctenochaetus binotatus* |
| Acanthuriformes | Acanthuridae | *Ctenochaetus striatus* |
| Acanthuriformes | Acanthuridae | *Naso annulatus* |
| Acanthuriformes | Acanthuridae | *Naso brevirostris* |
| Acanthuriformes | Acanthuridae | *Naso lituratus* |
| Acanthuriformes | Acanthuridae | *Naso unicornis* |
| Acanthuriformes | Acanthuridae | *Naso vlamingii* |
| Acanthuriformes | Acanthuridae | *Zebrasoma scopas* |
| Acanthuriformes | Acanthuridae | *Zebrasoma velifer* |
| Acanthuriformes | Zanclidae | *Zanclus cornutus* |
| Anguilliformes | Congridae | *Conger cinereus* |
| Anguilliformes | Muraenidae | *Gymnothorax pictus* |
| Atheriniformes | Atherinidae | *Craterocephalus mugiloides* |
| Atheriniformes | Melanotaeniidae | *Pseudomugil signifer* |
| Aulopiformes | Synodontidae | *Synodus dermatogenys* |
| Aulopiformes | Synodontidae | *Synodus variegatus* |
| Beloniformes | Belonidae | *Tylosurus crocodilus** |
| Beloniformes | Exocoetidae | *Exocoetus monocirrhus* |
| Beloniformes | Hemiramphidae | *Hyporhamphus quoyi* |
| Beloniformes | Zenarchopteridae | *Zenarchopterus dispar* |
| Blenniiformes | Blenniidae | *Atrosalarias fuscus* |
| Blenniiformes | Blenniidae | *Blenniella paula* |
| Blenniiformes | Blenniidae | *Cirripectes stigmaticus* |
| Blenniiformes | Blenniidae | *Crossosalarias macrospilus* |
| Blenniiformes | Blenniidae | *Ecsenius bicolor* |
| Blenniiformes | Blenniidae | *Ecsenius stictus* |
| Blenniiformes | Blenniidae | *Istiblennius edentulus* |
| Blenniiformes | Blenniidae | *Meiacanthus atrodorsalis* |
| Blenniiformes | Blenniidae | *Petroscirtes fallax* |
| Blenniiformes | Blenniidae | *Petroscirtes mitratus* |
| Blenniiformes | Blenniidae | *Petroscirtes variabilis* |
| Blenniiformes | Blenniidae | *Plagiotremus laudandus* |
| Blenniiformes | Blenniidae | *Plagiotremus tapeinosoma* |
| Blenniiformes | Blenniidae | *Salarias alboguttatus* |
| Blenniiformes | Blenniidae | *Salarias fasciatus* |
| Blenniiformes | Blenniidae | *Salarias guttatus* |
| Blenniiformes | Blenniidae | *Salarias* sp*.* |
| Blenniiformes | Tripterygiidae | *Enneapterygius elegans* |
| Blenniiformes | Tripterygiidae | *Enneapterygius hemimelas* |
| Blenniiformes | Tripterygiidae | *Enneapterygius philippinus* |
| Blenniiformes | Tripterygiidae | *Enneapterygius* sp*.** |
| Blenniiformes | Tripterygiidae | *Norfolkia thomasi* |
| Blenniiformes | Tripterygiidae | *Ucla xenogrammus* |
| Callionymiformes | Callionymidae | *Diplogrammus goramensis* |
| Carangiformes | Carangidae | *Caranx melampygus* |
| Carangiformes | Carangidae | *Caranx papuensis* |
| Carangiformes | Carangidae | *Scomberoides lysan* |
| Carangiformes | Carangidae | *Trachinotus baillonii* |
| Carangiformes | Carangidae | *Trachinotus blochii* |
| Carcharhiniformes | Carcharhinidae | *Carcharhinus amblyrhynchos* |
| Carcharhiniformes | Carcharhinidae | *Carcharhinus melanopterus* |
| Clupeiformes | Clupeidae | *Spratelloides gracilis* |
| Gobiiformes | Gobiidae | *Amblyeleotris wheeleri* |
| Gobiiformes | Gobiidae | *Amblygobius decussatus* |
| Gobiiformes | Gobiidae | *Amblygobius phalaena* |
| Gobiiformes | Gobiidae | *Amblygobius rainfordi* |
| Gobiiformes | Gobiidae | *Asterropteryx semipunctata* |
| Gobiiformes | Gobiidae | *Bathygobius coalitus* |
| Gobiiformes | Gobiidae | *Bathygobius cyclopterus** |
| Gobiiformes | Gobiidae | *Bathygobius* sp*.** |
| Gobiiformes | Gobiidae | *Bryaninops isis** |
| Gobiiformes | Gobiidae | *Callogobius sclateri* |
| Gobiiformes | Gobiidae | *Cryptocentrus leptocephalus* |
| Gobiiformes | Gobiidae | *Eviota guttata* |
| Gobiiformes | Gobiidae | *Eviota nebulosa* |
| Gobiiformes | Gobiidae | *Eviota nigriventris*^1^ |
| Gobiiformes | Gobiidae | *Eviota prasites* |
| Gobiiformes | Gobiidae | *Eviota queenslandica* |
| Gobiiformes | Gobiidae | *Eviota* sp*.* |
| Gobiiformes | Gobiidae | *Eviota* sp. 1 |
| Gobiiformes | Gobiidae | *Eviota* sp. 2 |
| Gobiiformes | Gobiidae | *Eviota* sp. 3 |
| Gobiiformes | Gobiidae | *Eviota* sp. 5 |
| Gobiiformes | Gobiidae | *Fusigobius duospilus* |
| Gobiiformes | Gobiidae | *Fusigobius neophytus* |
| Gobiiformes | Gobiidae | *Fusigobius signipinnis* |
| Gobiiformes | Gobiidae | *Gobiodon brochus* |
| Gobiiformes | Gobiidae | *Gobiodon ceramensis* |
| Gobiiformes | Gobiidae | *Gobiodon citrinus* |
| Gobiiformes | Gobiidae | *Gobiodon heterospilos* |
| Gobiiformes | Gobiidae | *Gobiodon oculolineatus* |
| Gobiiformes | Gobiidae | *Gobiodon okinawae* |
| Gobiiformes | Gobiidae | *Gobiodon quinquestrigatus* |
| Gobiiformes | Gobiidae | *Gobiodon rivulatus* |
| Gobiiformes | Gobiidae | *Gobiodon* sp. |
| Gobiiformes | Gobiidae | *Istigobius decoratus* |
| Gobiiformes | Gobiidae | *Istigobius ornatus* |
| Gobiiformes | Gobiidae | *Istigobius rigilius* |
| Gobiiformes | Gobiidae | *Macrodontogobius wilburi* |
| Gobiiformes | Gobiidae | *Paragobiodon echinocephalus* |
| Gobiiformes | Gobiidae | *Paragobiodon lacunicolus* |
| Gobiiformes | Gobiidae | *Paragobiodon modestus* |
| Gobiiformes | Gobiidae | *Paragobiodon* sp. |
| Gobiiformes | Gobiidae | *Paragobiodon xanthosomus* |
| Gobiiformes | Gobiidae | *Pleurosicya mossambica* |
| Gobiiformes | Gobiidae | *Priolepis inhaca* |
| Gobiiformes | Gobiidae | *Ptereleotris evides* |
| Gobiiformes | Gobiidae | *Trimma annosum* |
| Gobiiformes | Gobiidae | *Trimma benjamini* |
| Gobiiformes | Gobiidae | *Trimma caesiura** |
| Gobiiformes | Gobiidae | *Trimma emeryi* |
| Gobiiformes | Gobiidae | *Trimma necopinum* |
| Gobiiformes | Gobiidae | *Trimma* oki group 8 |
| Gobiiformes | Gobiidae | *Trimma* sp.*** |
| Gobiiformes | Gobiidae | *Trimma striatum* |
| Gobiiformes | Gobiidae | *Trimmatom* sp. |
| Gobiiformes | Gobiidae | *Valenciennea strigata* |
| Holocentriformes | Holocentridae | *Myripristis berndti* |
| Holocentriformes | Holocentridae | *Myripristis murdjan* |
| Holocentriformes | Holocentridae | *Neoniphon sammara* |
| Holocentriformes | Holocentridae | *Sargocentron caudimaculatum* |
| Holocentriformes | Holocentridae | *Sargocentron spiniferum* |
| Istiophoriformes | Sphyraenidae | *Sphyraena qenie* |
| Kurtiformes | Apogonidae | *Apogon crassiceps* |
| Kurtiformes | Apogonidae | *Apogon seminigracaudus* |
| Kurtiformes | Apogonidae | *Apogonichthyoides melas* |
| Kurtiformes | Apogonidae | *Cheilodipterus artus* |
| Kurtiformes | Apogonidae | *Cheilodipterus intermedius* |
| Kurtiformes | Apogonidae | *Cheilodipterus macrodon* |
| Kurtiformes | Apogonidae | *Cheilodipterus quinquelineatus* |
| Kurtiformes | Apogonidae | *Cheilodipterus* cf. *quinquelineatus* |
| Kurtiformes | Apogonidae | *Fibramia lateralis* |
| Kurtiformes | Apogonidae | *Fibramia thermalis* |
| Kurtiformes | Apogonidae | *Fowleria vaiulae* |
| Kurtiformes | Apogonidae | *Nectamia luxuria* |
| Kurtiformes | Apogonidae | *Nectamia savayensis* |
| Kurtiformes | Apogonidae | *Nectamia similis* |
| Kurtiformes | Apogonidae | *Nectamia viria* |
| Kurtiformes | Apogonidae | *Ostorhinchus compressus* |
| Kurtiformes | Apogonidae | *Ostorhinchus cookii* |
| Kurtiformes | Apogonidae | *Ostorhinchus cyanosoma* |
| Kurtiformes | Apogonidae | *Ostorhinchus doederleini* |
| Kurtiformes | Apogonidae | *Ostorhinchus nigrofasciatus* |
| Kurtiformes | Apogonidae | *Ostorhinchus properuptus* |
| Kurtiformes | Apogonidae | *Pristiapogon exostigma* |
| Kurtiformes | Apogonidae | *Pristiapogon kallopterus* |
| Kurtiformes | Apogonidae | *Pristicon trimaculatus* |
| Kurtiformes | Apogonidae | *Taeniamia fucata* |
| Kurtiformes | Apogonidae | *Zoramia leptacantha* |
| Kurtiformes | Apogonidae | *Zoramia viridiventer* |
| Labriformes | Labridae | *Anampses neoguinaicus* |
| Labriformes | Labridae | *Bodianus axillaris* |
| Labriformes | Labridae | *Cheilinus chlorourus* |
| Labriformes | Labridae | *Cheilinus fasciatus* |
| Labriformes | Labridae | *Cheilinus trilobatus* |
| Labriformes | Labridae | *Cirrhilabrus exquisitus** |
| Labriformes | Labridae | *Cirrhilabrus punctatus* |
| Labriformes | Labridae | *Coris batuensis* |
| Labriformes | Labridae | *Coris gaimard* |
| Labriformes | Labridae | *Epibulus insidiator* |
| Labriformes | Labridae | *Gomphosus varius* |
| Labriformes | Labridae | *Halichoeres chloropterus* |
| Labriformes | Labridae | *Halichoeres marginatus* |
| Labriformes | Labridae | *Halichoeres melanurus* |
| Labriformes | Labridae | *Halichoeres miniatus* |
| Labriformes | Labridae | *Halichoeres prosopeion* |
| Labriformes | Labridae | *Halichoeres scapularis* |
| Labriformes | Labridae | *Halichoeres trimaculatus* |
| Labriformes | Labridae | *Hemigymnus fasciatus* |
| Labriformes | Labridae | *Hemigymnus melapterus* |
| Labriformes | Labridae | *Labrichthys unilineatus* |
| Labriformes | Labridae | *Labroides dimidiatus* |
| Labriformes | Labridae | *Novaculichthys taeniourus* |
| Labriformes | Labridae | *Oxycheilinus digramma* |
| Labriformes | Labridae | *Pseudocheilinus hexataenia* |
| Labriformes | Labridae | *Pteragogus cryptus* |
| Labriformes | Labridae | *Stethojulis bandanensis* |
| Labriformes | Labridae | *Stethojulis* sp.*** |
| Labriformes | Labridae | *Stethojulis strigiventer* |
| Labriformes | Labridae | *Stethojulis trilineata* |
| Labriformes | Labridae | *Thalassoma amblycephalum* |
| Labriformes | Labridae | *Thalassoma hardwicke* |
| Labriformes | Labridae | *Thalassoma lunare* |
| Labriformes | Labridae | *Thalassoma lutescens* |
| Labriformes | Labridae | *Thalassoma nigrofasciatum* |
| Labriformes | Labridae | Labridae species* |
| Labriformes | Scaridae | *Chlorurus sordidus* |
| Labriformes | Scaridae | *Scarus flavipectoralis* |
| Labriformes | Scaridae | *Scarus frenatus* |
| Labriformes | Scaridae | *Scarus ghobban** |
| Labriformes | Scaridae | *Scarus niger* |
| Labriformes | Scaridae | *Scarus schlegeli* |
| Labriformes | Scaridae | *Scarus* sp. |
| Labriformes | Scaridae | *Scarus spinus* |
| Lophiiformes | Antennariidae | *Antennatus coccineus* |
| Moroniformes | Ephippidae | *Platax pinnatus* |
| Mugiliformes | Mugilidae | *Crenimugil crenilabis* |
| Mugiliformes | Mugilidae | *Ellochelon vaigiensis* |
| Mugiliformes | Mugilidae | *Moolgarda seheli* |
| Myliobatiformes | Dasyatidae | *Neotrygon trigonoides* |
| Myliobatiformes | Dasyatidae | *Taeniura lymma* |
| Orectolobiformes | Hemiscylliidae | *Hemiscyllium ocellatum** |
| Subseries Ovalentaria | Eleotridae | *Ophiocara porocephala* |
| Subseries Ovalentaria | Plesiopidae | *Assessor macneilli* |
| Subseries Ovalentaria | Plesiopidae | *Plesiops coeruleolineatus* |
| Subseries Ovalentaria | Plesiopidae | *Plesiops verecundus* |
| Subseries Ovalentaria | Pomacentridae | *Abudefduf septemfasciatus* |
| Subseries Ovalentaria | Pomacentridae | *Abudefduf sexfasciatus* |
| Subseries Ovalentaria | Pomacentridae | *Abudefduf sordidus* |
| Subseries Ovalentaria | Pomacentridae | *Abudefduf whitleyi* |
| Subseries Ovalentaria | Pomacentridae | *Acanthochromis polyacanthus* |
| Subseries Ovalentaria | Pomacentridae | *Amblyglyphidodon aureus* |
| Subseries Ovalentaria | Pomacentridae | *Amblyglyphidodon curacao* |
| Subseries Ovalentaria | Pomacentridae | *Amblyglyphidodon leucogaster* |
| Subseries Ovalentaria | Pomacentridae | *Amphiprion akindynos* |
| Subseries Ovalentaria | Pomacentridae | *Amphiprion melanopus* |
| Subseries Ovalentaria | Pomacentridae | *Chromis amboinensis** |
| Subseries Ovalentaria | Pomacentridae | *Chromis atripectoralis* |
| Subseries Ovalentaria | Pomacentridae | *Chromis ternatensis* |
| Subseries Ovalentaria | Pomacentridae | *Chromis viridis* |
| Subseries Ovalentaria | Pomacentridae | *Chromis weberi* |
| Subseries Ovalentaria | Pomacentridae | *Chrysiptera biocellata* |
| Subseries Ovalentaria | Pomacentridae | *Chrysiptera caesifrons* |
| Subseries Ovalentaria | Pomacentridae | *Chrysiptera cyanea* |
| Subseries Ovalentaria | Pomacentridae | *Chrysiptera flavipinnis* |
| Subseries Ovalentaria | Pomacentridae | *Chrysiptera rollandi* |
| Subseries Ovalentaria | Pomacentridae | *Chrysiptera talboti* |
| Subseries Ovalentaria | Pomacentridae | *Chrysiptera taupou* |
| Subseries Ovalentaria | Pomacentridae | *Dascyllus aruanus* |
| Subseries Ovalentaria | Pomacentridae | *Dascyllus reticulatus* |
| Subseries Ovalentaria | Pomacentridae | *Dischistodus melanotus* |
| Subseries Ovalentaria | Pomacentridae | *Dischistodus perspicillatus* |
| Subseries Ovalentaria | Pomacentridae | *Dischistodus prosopotaenia* |
| Subseries Ovalentaria | Pomacentridae | *Dischistodus pseudochrysopoecilus* |
| Subseries Ovalentaria | Pomacentridae | *Hemiglyphidodon plagiometopon* |
| Subseries Ovalentaria | Pomacentridae | *Neoglyphidodon melas* |
| Subseries Ovalentaria | Pomacentridae | *Neoglyphidodon nigroris* |
| Subseries Ovalentaria | Pomacentridae | *Neopomacentrus azysron* |
| Subseries Ovalentaria | Pomacentridae | *Neopomacentrus cyanomos* |
| Subseries Ovalentaria | Pomacentridae | *Plectroglyphidodon dickii** |
| Subseries Ovalentaria | Pomacentridae | *Plectroglyphidodon lacrymatus* |
| Subseries Ovalentaria | Pomacentridae | *Pomacentrus amboinensis* |
| Subseries Ovalentaria | Pomacentridae | *Pomacentrus bankanensis* |
| Subseries Ovalentaria | Pomacentridae | *Pomacentrus brachialis* |
| Subseries Ovalentaria | Pomacentridae | *Pomacentrus chrysurus* |
| Subseries Ovalentaria | Pomacentridae | *Pomacentrus coelestis* |
| Subseries Ovalentaria | Pomacentridae | *Pomacentrus lepidogenys* |
| Subseries Ovalentaria | Pomacentridae | *Pomacentrus moluccensis* |
| Subseries Ovalentaria | Pomacentridae | *Pomacentrus nagasakiensis* |
| Subseries Ovalentaria | Pomacentridae | *Pomacentrus reidi* |
| Subseries Ovalentaria | Pomacentridae | *Pomacentrus tripunctatus* |
| Subseries Ovalentaria | Pomacentridae | *Pomacentrus wardi* |
| Subseries Ovalentaria | Pomacentridae | *Premnas biaculeatus* |
| Subseries Ovalentaria | Pomacentridae | *Stegastes apicalis* |
| Subseries Ovalentaria | Pomacentridae | *Stegastes nigricans* |
| Subseries Ovalentaria | Pseudochromidae | *Cypho purpurascens* |
| Subseries Ovalentaria | Pseudochromidae | *Oxycercichthys veliferus* |
| Subseries Ovalentaria | Pseudochromidae | *Pictichromis coralensis** |
| Subseries Ovalentaria | Pseudochromidae | *Pictichromis paccagnellae* |
| Subseries Ovalentaria | Pseudochromidae | *Pseudochromis flammicauda* |
| Subseries Ovalentaria | Pseudochromidae | *Pseudochromis fuscus* |
| Subseries Ovalentaria | Pseudochromidae | *Pseudoplesiops sp.** |
| Subseries Ovalentaria | Pseudochromidae | Pseudochromidae species* |
| Perciformes | Caesionidae | *Caesio caerulaurea* |
| Perciformes | Caesionidae | *Caesio cuning* |
| Perciformes | Chaetodontidae | *Chaetodon aureofasciatus* |
| Perciformes | Chaetodontidae | *Chaetodon auriga* |
| Perciformes | Chaetodontidae | *Chaetodon baronessa* |
| Perciformes | Chaetodontidae | *Chaetodon bennetti* |
| Perciformes | Chaetodontidae | *Chaetodon citrinellus* |
| Perciformes | Chaetodontidae | *Chaetodon ephippium* |
| Perciformes | Chaetodontidae | *Chaetodon flavirostris* |
| Perciformes | Chaetodontidae | *Chaetodon kleinii* |
| Perciformes | Chaetodontidae | *Chaetodon lunula* |
| Perciformes | Chaetodontidae | *Chaetodon lunulatus* |
| Perciformes | Chaetodontidae | *Chaetodon ornatissimus** |
| Perciformes | Chaetodontidae | *Chaetodon pelewensis* |
| Perciformes | Chaetodontidae | *Chaetodon plebeius* |
| Perciformes | Chaetodontidae | *Chaetodon rafflesii* |
| Perciformes | Chaetodontidae | *Chaetodon rainfordi* |
| Perciformes | Chaetodontidae | *Chaetodon speculum* |
| Perciformes | Chaetodontidae | *Chaetodon trifascialis* |
| Perciformes | Chaetodontidae | *Chaetodon ulietensis* |
| Perciformes | Chaetodontidae | *Chaetodon unimaculatus* |
| Perciformes | Chaetodontidae | *Chaetodon vagabundus* |
| Perciformes | Chaetodontidae | *Chelmon rostratus* |
| Perciformes | Chaetodontidae | *Coradion chrysozonus* |
| Perciformes | Chaetodontidae | *Forcipiger flavissimus* |
| Perciformes | Chaetodontidae | *Heniochus chrysostomus* |
| Perciformes | Chaetodontidae | *Heniochus varius* |
| Perciformes | Cirrhitidae | *Paracirrhites forsteri* |
| Perciformes | Gerreidae | *Gerres erythrourus* |
| Perciformes | Gerreidae | *Gerres longirostris* |
| Perciformes | Gerreidae | *Gerres oyena* |
| Perciformes | Haemulidae | *Plectorhinchus albovittatus* |
| Perciformes | Haemulidae | *Plectorhinchus chaetodonoides* |
| Perciformes | Haemulidae | *Plectorhinchus chrysotaenia* |
| Perciformes | Haemulidae | *Plectorhinchus flavomaculatus* |
| Perciformes | Haemulidae | *Plectorhinchus lessonii* |
| Perciformes | Haemulidae | *Plectorhinchus lineatus* |
| Perciformes | Haemulidae | *Plectorhinchus unicolor* |
| Perciformes | Kyphosidae | *Kyphosus cinerascens* |
| Perciformes | Kyphosidae | *Kyphosus vaigiensis* |
| Perciformes | Lutjanidae | *Aprion virescens* |
| Perciformes | Lutjanidae | *Lutjanus argentimaculatus* |
| Perciformes | Lutjanidae | *Lutjanus bohar* |
| Perciformes | Lutjanidae | *Lutjanus carponotatus* |
| Perciformes | Lutjanidae | *Lutjanus ehrenbergii* |
| Perciformes | Lutjanidae | *Lutjanus fulviflamma* |
| Perciformes | Lutjanidae | *Lutjanus monostigma* |
| Perciformes | Lutjanidae | *Lutjanus russellii* |
| Perciformes | Lutjanidae | *Lutjanus vitta* |
| Perciformes | Lutjanidae | *Macolor niger* |
| Perciformes | Mullidae | *Parupeneus barberinus* |
| Perciformes | Mullidae | *Parupeneus ciliatus* |
| Perciformes | Mullidae | *Parupeneus crassilabris* |
| Perciformes | Mullidae | *Parupeneus indicus* |
| Perciformes | Mullidae | *Parupeneus multifasciatus* |
| Perciformes | Pomacanthidae | *Centropyge bicolor* |
| Perciformes | Pomacanthidae | *Centropyge bispinosa* |
| Perciformes | Pomacanthidae | *Centropyge vrolikii* |
| Perciformes | Pomacanthidae | *Pomacanthus imperator** |
| Perciformes | Pomacanthidae | *Pomacanthus sexstriatus* |
| Perciformes | Pomacanthidae | *Pygoplites diacanthus* |
| Perciformes | Priacanthidae | *Priacanthus hamrur* |
| Perciformes | Serranidae | *Cephalopholis argus** |
| Perciformes | Serranidae | *Cephalopholis boenak* |
| Perciformes | Serranidae | *Cephalopholis cyanostigma* |
| Perciformes | Serranidae | *Cephalopholis microprion* |
| Perciformes | Serranidae | *Diploprion bifasciatum* |
| Perciformes | Serranidae | *Epinephelus maculatus* |
| Perciformes | Serranidae | *Epinephelus malabaricus* |
| Perciformes | Serranidae | *Epinephelus ongus* |
| Perciformes | Serranidae | *Plectropomus laevis* |
| Perciformes | Serranidae | *Plectropomus leopardus* |
| Perciformes | Serranidae | *Pseudogramma polyacantha* |
| Perciformes | Siganidae | *Siganus corallinus* |
| Perciformes | Siganidae | *Siganus doliatus* |
| Perciformes | Siganidae | *Siganus lineatus* |
| Perciformes | Siganidae | *Siganus punctatissimus* |
| Perciformes | Siganidae | *Siganus punctatus* |
| Perciformes | Siganidae | *Siganus vulpinus* |
| Perciformes | Terapontidae | *Amniataba caudavittata* |
| Perciformes | Terapontidae | *Terapon jarbua* |
| Pleuronectiformes | Bothidae | *Bothus pantherinus* |
| Scombriformes | Scombridae | *Grammatorcynus bicarinatus* |
| Scombriformes | Scombridae | *Scomberomorus commerson* |
| Scorpaeniformes | Platycephalidae | *Onigocia sibogae* |
| Scorpaeniformes | Platycephalidae | *Platycephalus westraliae* |
| Scorpaeniformes | Platycephalidae | *Sunagocia arenicola* |
| Scorpaeniformes | Scorpaenidae | *Caracanthus unipinna** |
| Scorpaeniformes | Scorpaenidae | *Dendrochirus zebra* |
| Scorpaeniformes | Scorpaenidae | *Parascorpaena mossambica* |
| Scorpaeniformes | Scorpaenidae | *Pterois volitans* |
| Scorpaeniformes | Scorpaenidae | *Scorpaena* sp. |
| Scorpaeniformes | Scorpaenidae | *Scorpaenopsis brevifrons** |
| Scorpaeniformes | Scorpaenidae | *Scorpaenopsis venosa* |
| Scorpaeniformes | Scorpaenidae | *Sebastapistes strongia* |
| Spariformes | Lethrinidae | *Lethrinus atkinsoni* |
| Spariformes | Lethrinidae | *Lethrinus harak* |
| Spariformes | Lethrinidae | *Lethrinus obsoletus* |
| Spariformes | Lethrinidae | *Lethrinus ravus* |
| Spariformes | Lethrinidae | *Lethrinus semicinctus* |
| Spariformes | Lethrinidae | *Lethrinus xanthochilus* |
| Spariformes | Lethrinidae | *Monotaxis heterodon* |
| Spariformes | Nemipteridae | *Scolopsis bilineata* |
| Spariformes | Nemipteridae | *Scolopsis lineata* |
| Spariformes | Nemipteridae | *Scolopsis margaritifera* |
| Spariformes | Nemipteridae | *Scolopsis monogramma* |
| Spariformes | Sillaginidae | *Sillago ciliata* |
| Syngnathiformes | Aulostomidae | *Aulostomus chinensis* |
| Syngnathiformes | Centriscidae | *Aeoliscus strigatus* |
| Syngnathiformes | Fistulariidae | *Fistularia commersonii** |
| Syngnathiformes | Syngnathidae | *Corythoichthys amplexus* |
| Syngnathiformes | Syngnathidae | *Doryrhamphus melanopleura* |
| Syngnathiformes | Syngnathidae | *Halicampus nitidus* |
| Tetraodontiformes | Balistidae | *Abalistes stellatus* |
| Tetraodontiformes | Balistidae | *Balistapus undulatus* |
| Tetraodontiformes | Balistidae | *Balistoides viridescens* |
| Tetraodontiformes | Balistidae | *Pseudobalistes fuscus* |
| Tetraodontiformes | Balistidae | *Rhinecanthus aculeatus* |
| Tetraodontiformes | Balistidae | *Sufflamen chrysopterum* |
| Tetraodontiformes | Diodontidae | *Diodon hystrix* |
| Tetraodontiformes | Monacanthidae | *Cantherhines pardalis* |
| Tetraodontiformes | Monacanthidae | *Oxymonacanthus longirostris* |
| Tetraodontiformes | Monacanthidae | *Paraluteres prionurus* |
| Tetraodontiformes | Monacanthidae | *Pervagor janthinosoma* |
| Tetraodontiformes | Monacanthidae | *Rudarius minutus* |
| Tetraodontiformes | Ostraciidae | *Ostracion cubicus* |
| Tetraodontiformes | Ostraciidae | *Ostracion meleagris* |
| Tetraodontiformes | Tetraodontidae | *Arothron hispidus* |
| Tetraodontiformes | Tetraodontidae | *Arothron manilensis* |
| Tetraodontiformes | Tetraodontidae | *Arothron nigropunctatus* |
| Tetraodontiformes | Tetraodontidae | *Canthigaster bennetti* |
| Tetraodontiformes | Tetraodontidae | *Canthigaster solandri* |
| Tetraodontiformes | Tetraodontidae | *Canthigaster* sp.*** |
| Tetraodontiformes | Tetraodontidae | *Canthigaster valentini* |
| Trachiniformes | Pinguipedidae | *Parapercis australis* |
| Trachiniformes | Pinguipedidae | *Parapercis queenslandica* |
|  |  |  |

^1^ very likely part of a species-complex (Greenfield and Winterbottom 2016)
