# Supplementary material for: DNA barcoding the fishes of Lizard Island (Great Barrier Reef)
Source: Biodivers Data J. 2017 Apr 13;(5):e12409. doi: 10.3897/BDJ.5.e12409 (PMC5515069; doi:10.3897/BDJ.5.e12409)
Supplement: Supplementary material 3 — Neighbour Joining tree based on K2P distances [file bdj-05-e12409-s003.pdf]

BOLD TaxonID Tree

Title	:	Tree Result - Search (983 records)
Date	:	12-Dec-2016
Data Type	:	Nucleotide
Distance Model	:	Kimura 2 Parameter
Marker	:	COI-5P
Colourization	:	Taxonomy: Family
Label	:	Process ID
Label	:	Family
Label	:	Taxon
Label	:	Barcode Cluster (BIN)
Sequence Count	:	983
Species count	:	370
Genus count	:	176
Family count	:	59
Unidentified	:	3
BIN Count	:	374

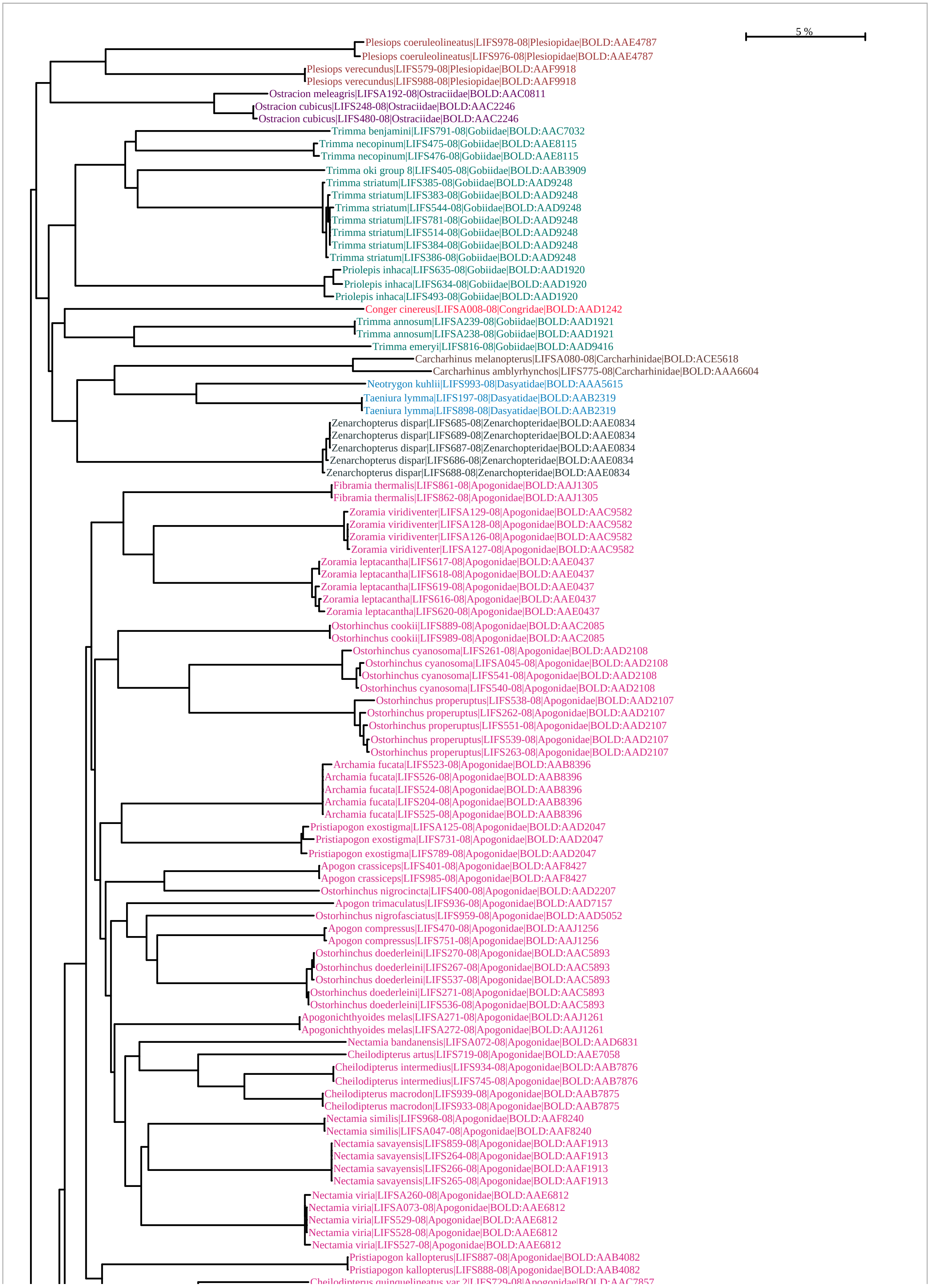
5 %
Plesiops coeruleolineatus|LIFS978-08|Plesiopidae|BOLD:AAE4787
Plesiops coeruleolineatus|LIFS976-08|Plesiopidae|BOLD:AAE4787
Plesiops verecundus|LIFS579-08|Plesiopidae|BOLD:AAF9918
Plesiops verecundus|LIFS988-08|Plesiopidae|BOLD:AAF9918
Ostracion meleagris|LIFSA192-08|Ostraciidae|BOLD:AAC0811
Ostracion cubicus|LIFS248-08|Ostraciidae|BOLD:AAC2246
Ostracion cubicus|LIFS480-08|Ostraciidae|BOLD:AAC2246
Trimma benjamini|LIFS791-08|Gobiidae|BOLD:AAC7032
Trimma necopinum|LIFS475-08|Gobiidae|BOLD:AAE8115
Trimma necopinum|LIFS476-08|Gobiidae|BOLD:AAE8115
Trimma oki group 8|LIFS405-08|Gobiidae|BOLD:AAB3909
Trimma striatum|LIFS385-08|Gobiidae|BOLD:AAD9248
Trimma striatum|LIFS383-08|Gobiidae|BOLD:AAD9248
Trimma striatum|LIFS544-08|Gobiidae|BOLD:AAD9248
Trimma striatum|LIFS781-08|Gobiidae|BOLD:AAD9248
Trimma striatum|LIFS514-08|Gobiidae|BOLD:AAD9248
Trimma striatum|LIFS384-08|Gobiidae|BOLD:AAD9248
Trimma striatum|LIFS386-08|Gobiidae|BOLD:AAD9248
Priolepis inhaca|LIFS635-08|Gobiidae|BOLD:AAD1920
Priolepis inhaca|LIFS634-08|Gobiidae|BOLD:AAD1920
Priolepis inhaca|LIFS493-08|Gobiidae|BOLD:AAD1920
Conger cinereus|LIFSA008-08|Congridae|BOLD:AAD1242
Trimma annosum|LIFSA239-08|Gobiidae|BOLD:AAD1921
Trimma annosum|LIFSA238-08|Gobiidae|BOLD:AAD1921
Trimma emeryi|LIFS816-08|Gobiidae|BOLD:AAD9416
Carcharhinus melanopterus|LIFSA080-08|Carcharhinidae|BOLD:ACE5618
Carcharhinus amblyrhynchos|LIFS775-08|Carcharhinidae|BOLD:AAA6604
Neotrygon kuhlii|LIFS993-08|Dasyatidae|BOLD:AAA5615
Taeniura lymma|LIFS197-08|Dasyatidae|BOLD:AAB2319
Taeniura lymma|LIFS898-08|Dasyatidae|BOLD:AAB2319
Zenarchopterus dispar|LIFS685-08|Zenarchopteridae|BOLD:AAE0834
Zenarchopterus dispar|LIFS689-08|Zenarchopteridae|BOLD:AAE0834
Zenarchopterus dispar|LIFS687-08|Zenarchopteridae|BOLD:AAE0834
Zenarchopterus dispar|LIFS686-08|Zenarchopteridae|BOLD:AAE0834
Zenarchopterus dispar|LIFS688-08|Zenarchopteridae|BOLD:AAE0834
Fibramia thermalis|LIFS861-08|Apogonidae|BOLD:AAJ1305
Fibramia thermalis|LIFS862-08|Apogonidae|BOLD:AAJ1305
Zoramia viridiventer|LIFSA129-08|Apogonidae|BOLD:AAC9582
Zoramia viridiventer|LIFSA128-08|Apogonidae|BOLD:AAC9582
Zoramia viridiventer|LIFSA126-08|Apogonidae|BOLD:AAC9582
Zoramia viridiventer|LIFSA127-08|Apogonidae|BOLD:AAC9582
Zoramia leptacantha|LIFS617-08|Apogonidae|BOLD:AAE0437
Zoramia leptacantha|LIFS618-08|Apogonidae|BOLD:AAE0437
Zoramia leptacantha|LIFS619-08|Apogonidae|BOLD:AAE0437
Zoramia leptacantha|LIFS616-08|Apogonidae|BOLD:AAE0437
Zoramia leptacantha|LIFS620-08|Apogonidae|BOLD:AAE0437
Ostorhinchus cookii|LIFS889-08|Apogonidae|BOLD:AAC2085
Ostorhinchus cookii|LIFS989-08|Apogonidae|BOLD:AAC2085
Ostorhinchus cyanosoma|LIFS261-08|Apogonidae|BOLD:AAD2108
Ostorhinchus cyanosoma|LIFSA045-08|Apogonidae|BOLD:AAD2108
Ostorhinchus cyanosoma|LIFS541-08|Apogonidae|BOLD:AAD2108
Ostorhinchus cyanosoma|LIFS540-08|Apogonidae|BOLD:AAD2108
Ostorhinchus properuptus|LIFS538-08|Apogonidae|BOLD:AAD2107
Ostorhinchus properuptus|LIFS262-08|Apogonidae|BOLD:AAD2107
Ostorhinchus properuptus|LIFS551-08|Apogonidae|BOLD:AAD2107
Ostorhinchus properuptus|LIFS539-08|Apogonidae|BOLD:AAD2107
Ostorhinchus properuptus|LIFS263-08|Apogonidae|BOLD:AAD2107
Archamia fucata|LIFS523-08|Apogonidae|BOLD:AAB8396
Archamia fucata|LIFS526-08|Apogonidae|BOLD:AAB8396
Archamia fucata|LIFS524-08|Apogonidae|BOLD:AAB8396
Archamia fucata|LIFS204-08|Apogonidae|BOLD:AAB8396
Archamia fucata|LIFS525-08|Apogonidae|BOLD:AAB8396
Pristiapogon exostigma|LIFSA125-08|Apogonidae|BOLD:AAD2047
Pristiapogon exostigma|LIFS731-08|Apogonidae|BOLD:AAD2047
Pristiapogon exostigma|LIFS789-08|Apogonidae|BOLD:AAD2047
Apogon crassiceps|LIFS401-08|Apogonidae|BOLD:AAF8427
Apogon crassiceps|LIFS985-08|Apogonidae|BOLD:AAF8427
Ostorhinchus nigrocincta|LIFS400-08|Apogonidae|BOLD:AAD2207
Apogon trimaculatus|LIFS936-08|Apogonidae|BOLD:AAD7157
Ostorhinchus nigrofasciatus|LIFS959-08|Apogonidae|BOLD:AAD5052
Apogon compressus|LIFS470-08|Apogonidae|BOLD:AAJ1256
Apogon compressus|LIFS751-08|Apogonidae|BOLD:AAJ1256
Ostorhinchus doederleini|LIFS270-08|Apogonidae|BOLD:AAC5893
Ostorhinchus doederleini|LIFS267-08|Apogonidae|BOLD:AAC5893
Ostorhinchus doederleini|LIFS537-08|Apogonidae|BOLD:AAC5893
Ostorhinchus doederleini|LIFS271-08|Apogonidae|BOLD:AAC5893
Ostorhinchus doederleini|LIFS536-08|Apogonidae|BOLD:AAC5893
Apogonichthyoides melas|LIFSA271-08|Apogonidae|BOLD:AAJ1261
Apogonichthyoides melas|LIFSA272-08|Apogonidae|BOLD:AAJ1261
Nectamia bandanensis|LIFSA072-08|Apogonidae|BOLD:AAD6831
Cheilodipterus artus|LIFS719-08|Apogonidae|BOLD:AAE7058
Cheilodipterus intermedius|LIFS934-08|Apogonidae|BOLD:AAB7876
Cheilodipterus intermedius|LIFS745-08|Apogonidae|BOLD:AAB7876
Cheilodipterus macrodon|LIFS939-08|Apogonidae|BOLD:AAB7875
Cheilodipterus macrodon|LIFS933-08|Apogonidae|BOLD:AAB7875
Nectamia similis|LIFS968-08|Apogonidae|BOLD:AAF8240
Nectamia similis|LIFSA047-08|Apogonidae|BOLD:AAF8240
Nectamia savayensis|LIFS859-08|Apogonidae|BOLD:AAF1913
Nectamia savayensis|LIFS264-08|Apogonidae|BOLD:AAF1913
Nectamia savayensis|LIFS266-08|Apogonidae|BOLD:AAF1913
Nectamia savayensis|LIFS265-08|Apogonidae|BOLD:AAF1913
Nectamia viria|LIFSA260-08|Apogonidae|BOLD:AAE6812
Nectamia viria|LIFSA073-08|Apogonidae|BOLD:AAE6812
Nectamia viria|LIFS529-08|Apogonidae|BOLD:AAE6812
Nectamia viria|LIFS528-08|Apogonidae|BOLD:AAE6812
Nectamia viria|LIFS527-08|Apogonidae|BOLD:AAE6812
Pristiapogon kallopterus|LIFS887-08|Apogonidae|BOLD:AAB4082
Pristiapogon kallopterus|LIFS888-08|Apogonidae|BOLD:AAB4082
Cheilodipterus quinquelineatus var 2|LIFS729-08|Apogonidae|BOLD:AAC7857

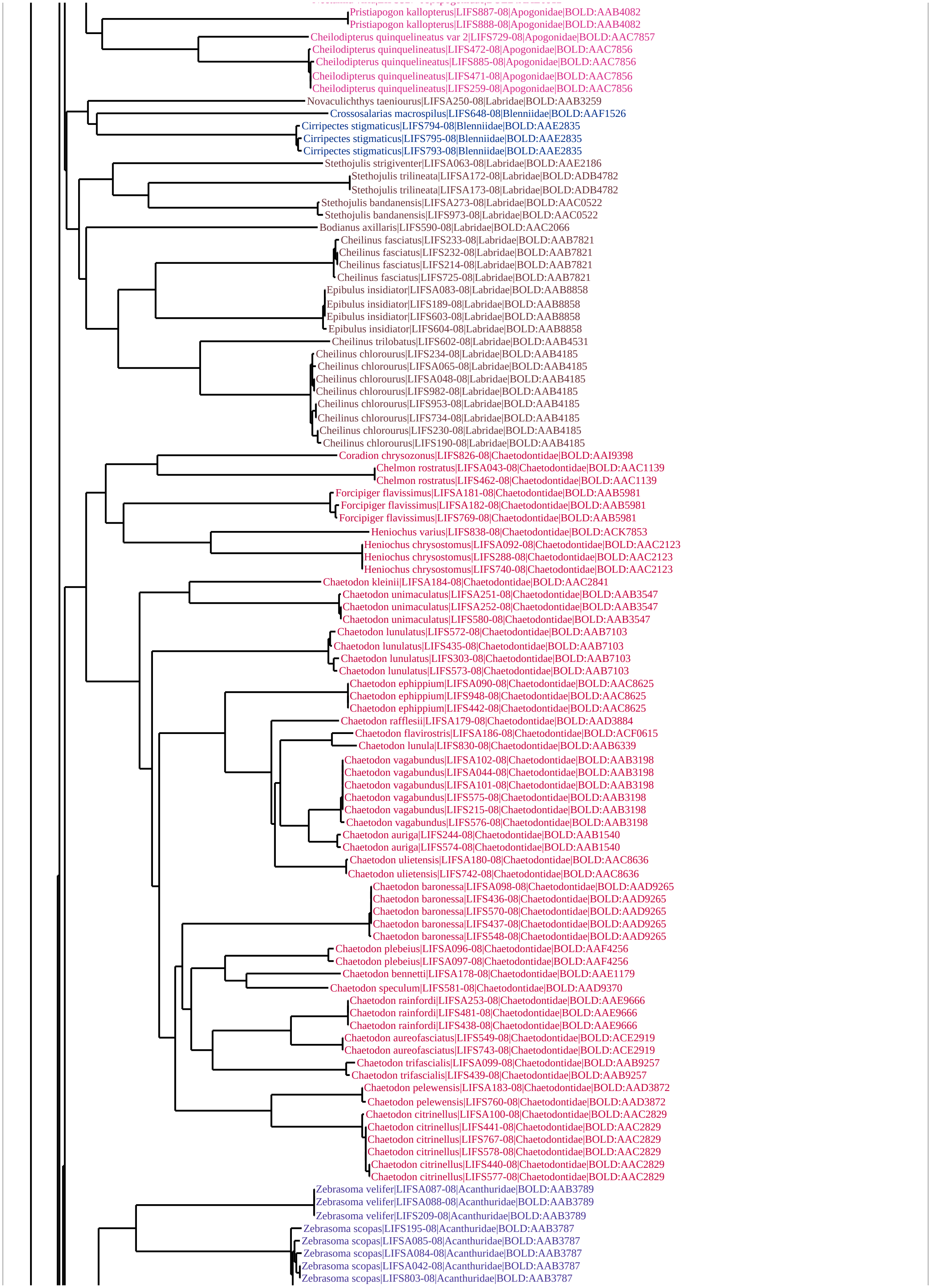
Pristiapogon kallopterus|LIFS887-08|Apogonidae|BOLD:AAB4082
Pristiapogon kallopterus|LIFS888-08|Apogonidae|BOLD:AAB4082
Cheilodipterus quinquelineatus var 2|LIFS729-08|Apogonidae|BOLD:AAC7857
Cheilodipterus quinquelineatus|LIFS472-08|Apogonidae|BOLD:AAC7856
Cheilodipterus quinquelineatus|LIFS885-08|Apogonidae|BOLD:AAC7856
Cheilodipterus quinquelineatus|LIFS471-08|Apogonidae|BOLD:AAC7856
Cheilodipterus quinquelineatus|LIFS259-08|Apogonidae|BOLD:AAC7856
Novaculichthys taeniourus|LIFSA250-08|Labridae|BOLD:AAB3259
Crossosalarias macrospilus|LIFS648-08|Blenniidae|BOLD:AAF1526
Cirripectes stigmaticus|LIFS794-08|Blenniidae|BOLD:AAE2835
Cirripectes stigmaticus|LIFS795-08|Blenniidae|BOLD:AAE2835
Cirripectes stigmaticus|LIFS793-08|Blenniidae|BOLD:AAE2835
Stethojulis strigiventer|LIFSA063-08|Labridae|BOLD:AAE2186
Stethojulis trilineata|LIFSA172-08|Labridae|BOLD:ADB4782
Stethojulis trilineata|LIFSA173-08|Labridae|BOLD:ADB4782
Stethojulis bandanensis|LIFSA273-08|Labridae|BOLD:AAC0522
Stethojulis bandanensis|LIFS973-08|Labridae|BOLD:AAC0522
Bodianus axillaris|LIFS590-08|Labridae|BOLD:AAC2066
Cheilinus fasciatus|LIFS233-08|Labridae|BOLD:AAB7821
Cheilinus fasciatus|LIFS232-08|Labridae|BOLD:AAB7821
Cheilinus fasciatus|LIFS214-08|Labridae|BOLD:AAB7821
Cheilinus fasciatus|LIFS725-08|Labridae|BOLD:AAB7821
Epibulus insidiator|LIFSA083-08|Labridae|BOLD:AAB8858
Epibulus insidiator|LIFS189-08|Labridae|BOLD:AAB8858
Epibulus insidiator|LIFS603-08|Labridae|BOLD:AAB8858
Epibulus insidiator|LIFS604-08|Labridae|BOLD:AAB8858
Cheilinus trilobatus|LIFS602-08|Labridae|BOLD:AAB4531
Cheilinus chlorourus|LIFS234-08|Labridae|BOLD:AAB4185
Cheilinus chlorourus|LIFSA065-08|Labridae|BOLD:AAB4185
Cheilinus chlorourus|LIFSA048-08|Labridae|BOLD:AAB4185
Cheilinus chlorourus|LIFS982-08|Labridae|BOLD:AAB4185
Cheilinus chlorourus|LIFS953-08|Labridae|BOLD:AAB4185
Cheilinus chlorourus|LIFS734-08|Labridae|BOLD:AAB4185
Cheilinus chlorourus|LIFS230-08|Labridae|BOLD:AAB4185
Cheilinus chlorourus|LIFS190-08|Labridae|BOLD:AAB4185
Coradion chrysozonus|LIFS826-08|Chaetodontidae|BOLD:AAI9398
Chelmon rostratus|LIFSA043-08|Chaetodontidae|BOLD:AAC1139
Chelmon rostratus|LIFS462-08|Chaetodontidae|BOLD:AAC1139
Forcipiger flavissimus|LIFSA181-08|Chaetodontidae|BOLD:AAB5981
Forcipiger flavissimus|LIFSA182-08|Chaetodontidae|BOLD:AAB5981
Forcipiger flavissimus|LIFS769-08|Chaetodontidae|BOLD:AAB5981
Heniochus varius|LIFS838-08|Chaetodontidae|BOLD:ACK7853
Heniochus chrysostomus|LIFSA092-08|Chaetodontidae|BOLD:AAC2123
Heniochus chrysostomus|LIFS288-08|Chaetodontidae|BOLD:AAC2123
Heniochus chrysostomus|LIFS740-08|Chaetodontidae|BOLD:AAC2123
Chaetodon kleinii|LIFSA184-08|Chaetodontidae|BOLD:AAC2841
Chaetodon unimaculatus|LIFSA251-08|Chaetodontidae|BOLD:AAB3547
Chaetodon unimaculatus|LIFSA252-08|Chaetodontidae|BOLD:AAB3547
Chaetodon unimaculatus|LIFS580-08|Chaetodontidae|BOLD:AAB3547
Chaetodon lunulatus|LIFS572-08|Chaetodontidae|BOLD:AAB7103
Chaetodon lunulatus|LIFS435-08|Chaetodontidae|BOLD:AAB7103
Chaetodon lunulatus|LIFS303-08|Chaetodontidae|BOLD:AAB7103
Chaetodon lunulatus|LIFS573-08|Chaetodontidae|BOLD:AAB7103
Chaetodon ephippium|LIFSA090-08|Chaetodontidae|BOLD:AAC8625
Chaetodon ephippium|LIFS948-08|Chaetodontidae|BOLD:AAC8625
Chaetodon ephippium|LIFS442-08|Chaetodontidae|BOLD:AAC8625
Chaetodon rafflesii|LIFSA179-08|Chaetodontidae|BOLD:AAD3884
Chaetodon flavirostris|LIFSA186-08|Chaetodontidae|BOLD:ACF0615
Chaetodon lunula|LIFS830-08|Chaetodontidae|BOLD:AAB6339
Chaetodon vagabundus|LIFSA102-08|Chaetodontidae|BOLD:AAB3198
Chaetodon vagabundus|LIFSA044-08|Chaetodontidae|BOLD:AAB3198
Chaetodon vagabundus|LIFSA101-08|Chaetodontidae|BOLD:AAB3198
Chaetodon vagabundus|LIFS575-08|Chaetodontidae|BOLD:AAB3198
Chaetodon vagabundus|LIFS215-08|Chaetodontidae|BOLD:AAB3198
Chaetodon vagabundus|LIFS576-08|Chaetodontidae|BOLD:AAB3198
Chaetodon auriga|LIFS244-08|Chaetodontidae|BOLD:AAB1540
Chaetodon auriga|LIFS574-08|Chaetodontidae|BOLD:AAB1540
Chaetodon ulietensis|LIFSA180-08|Chaetodontidae|BOLD:AAC8636
Chaetodon ulietensis|LIFS742-08|Chaetodontidae|BOLD:AAC8636
Chaetodon baronessa|LIFSA098-08|Chaetodontidae|BOLD:AAD9265
Chaetodon baronessa|LIFS436-08|Chaetodontidae|BOLD:AAD9265
Chaetodon baronessa|LIFS570-08|Chaetodontidae|BOLD:AAD9265
Chaetodon baronessa|LIFS437-08|Chaetodontidae|BOLD:AAD9265
Chaetodon baronessa|LIFS548-08|Chaetodontidae|BOLD:AAD9265
Chaetodon plebeius|LIFSA096-08|Chaetodontidae|BOLD:AAF4256
Chaetodon plebeius|LIFSA097-08|Chaetodontidae|BOLD:AAF4256
Chaetodon bennetti|LIFSA178-08|Chaetodontidae|BOLD:AAE1179
Chaetodon speculum|LIFS581-08|Chaetodontidae|BOLD:AAD9370
Chaetodon rainfordi|LIFSA253-08|Chaetodontidae|BOLD:AAE9666
Chaetodon rainfordi|LIFS481-08|Chaetodontidae|BOLD:AAE9666
Chaetodon rainfordi|LIFS438-08|Chaetodontidae|BOLD:AAE9666
Chaetodon aureofasciatus|LIFS549-08|Chaetodontidae|BOLD:ACE2919
Chaetodon aureofasciatus|LIFS743-08|Chaetodontidae|BOLD:ACE2919
Chaetodon trifascialis|LIFSA099-08|Chaetodontidae|BOLD:AAB9257
Chaetodon trifascialis|LIFS439-08|Chaetodontidae|BOLD:AAB9257
Chaetodon pelewensis|LIFSA183-08|Chaetodontidae|BOLD:AAD3872
Chaetodon pelewensis|LIFS760-08|Chaetodontidae|BOLD:AAD3872
Chaetodon citrinellus|LIFSA100-08|Chaetodontidae|BOLD:AAC2829
Chaetodon citrinellus|LIFS441-08|Chaetodontidae|BOLD:AAC2829
Chaetodon citrinellus|LIFS767-08|Chaetodontidae|BOLD:AAC2829
Chaetodon citrinellus|LIFS578-08|Chaetodontidae|BOLD:AAC2829
Chaetodon citrinellus|LIFS440-08|Chaetodontidae|BOLD:AAC2829
Chaetodon citrinellus|LIFS577-08|Chaetodontidae|BOLD:AAC2829
Zebrasoma velifer|LIFSA087-08|Acanthuridae|BOLD:AAB3789
Zebrasoma velifer|LIFSA088-08|Acanthuridae|BOLD:AAB3789
Zebrasoma velifer|LIFS209-08|Acanthuridae|BOLD:AAB3789
Zebrasoma scopas|LIFS195-08|Acanthuridae|BOLD:AAB3787
Zebrasoma scopas|LIFSA085-08|Acanthuridae|BOLD:AAB3787
Zebrasoma scopas|LIFSA084-08|Acanthuridae|BOLD:AAB3787
Zebrasoma scopas|LIFSA042-08|Acanthuridae|BOLD:AAB3787
Zebrasoma scopas|LIFS803-08|Acanthuridae|BOLD:AAB3787

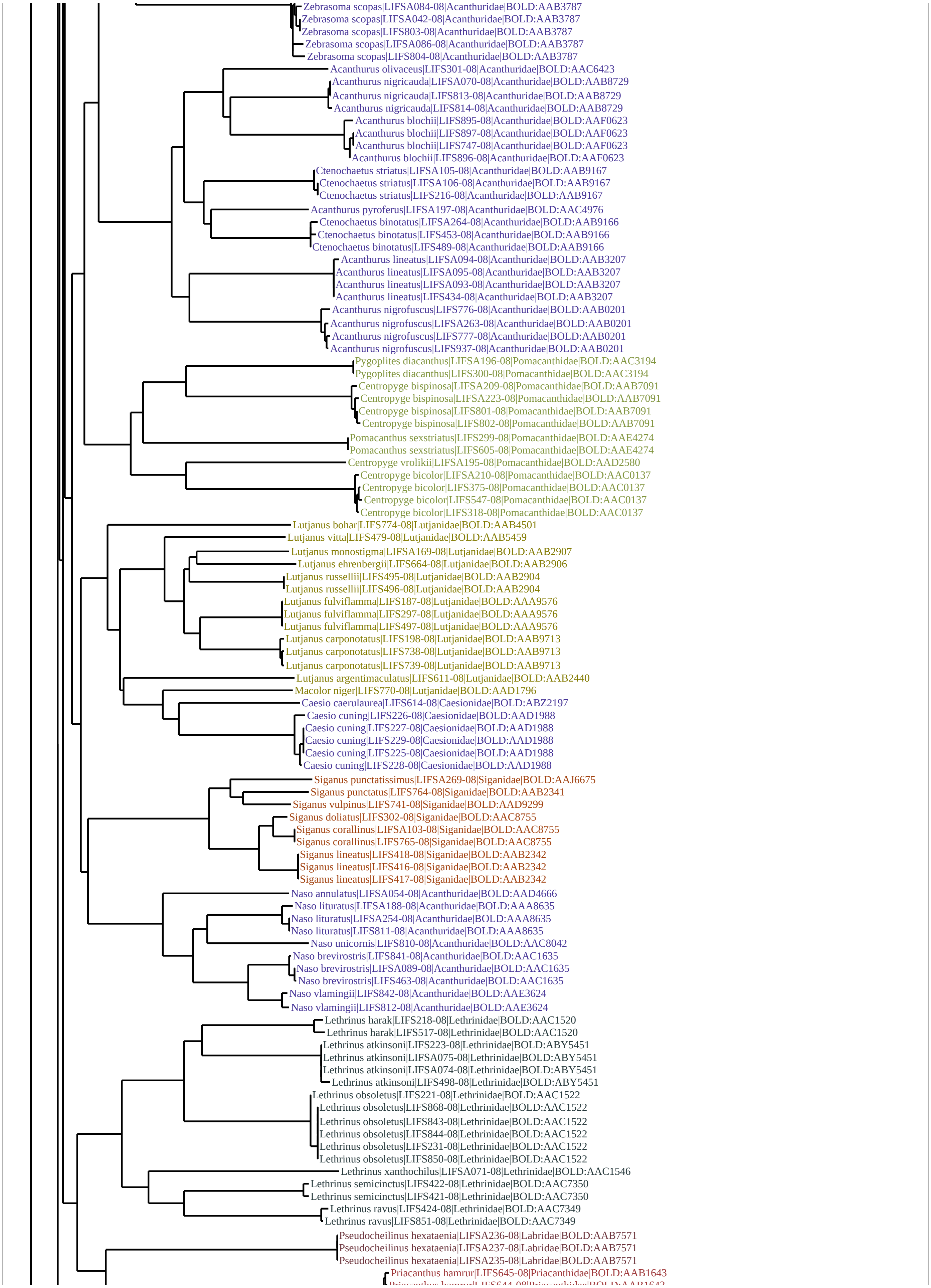
Zebrasoma scopas|LIFSA084-08|Acanthuridae|BOLD:AAB3787
Zebrasoma scopas|LIFSA042-08|Acanthuridae|BOLD:AAB3787
Zebrasoma scopas|LIFS803-08|Acanthuridae|BOLD:AAB3787
Zebrasoma scopas|LIFSA086-08|Acanthuridae|BOLD:AAB3787
Zebrasoma scopas|LIFS804-08|Acanthuridae|BOLD:AAB3787
Acanthurus olivaceus|LIFS301-08|Acanthuridae|BOLD:AAC6423
Acanthurus nigricauda|LIFSA070-08|Acanthuridae|BOLD:AAB8729
Acanthurus nigricauda|LIFS813-08|Acanthuridae|BOLD:AAB8729
Acanthurus nigricauda|LIFS814-08|Acanthuridae|BOLD:AAB8729
Acanthurus blochii|LIFS895-08|Acanthuridae|BOLD:AAF0623
Acanthurus blochii|LIFS897-08|Acanthuridae|BOLD:AAF0623
Acanthurus blochii|LIFS747-08|Acanthuridae|BOLD:AAF0623
Acanthurus blochii|LIFS896-08|Acanthuridae|BOLD:AAF0623
Ctenochaetus striatus|LIFSA105-08|Acanthuridae|BOLD:AAB9167
Ctenochaetus striatus|LIFSA106-08|Acanthuridae|BOLD:AAB9167
Ctenochaetus striatus|LIFS216-08|Acanthuridae|BOLD:AAB9167
Acanthurus pyroferus|LIFSA197-08|Acanthuridae|BOLD:AAC4976
Ctenochaetus binotatus|LIFSA264-08|Acanthuridae|BOLD:AAB9166
Ctenochaetus binotatus|LIFS453-08|Acanthuridae|BOLD:AAB9166
Ctenochaetus binotatus|LIFS489-08|Acanthuridae|BOLD:AAB9166
Acanthurus lineatus|LIFSA094-08|Acanthuridae|BOLD:AAB3207
Acanthurus lineatus|LIFSA095-08|Acanthuridae|BOLD:AAB3207
Acanthurus lineatus|LIFSA093-08|Acanthuridae|BOLD:AAB3207
Acanthurus lineatus|LIFS434-08|Acanthuridae|BOLD:AAB3207
Acanthurus nigrofuscus|LIFS776-08|Acanthuridae|BOLD:AAB0201
Acanthurus nigrofuscus|LIFSA263-08|Acanthuridae|BOLD:AAB0201
Acanthurus nigrofuscus|LIFS777-08|Acanthuridae|BOLD:AAB0201
Acanthurus nigrofuscus|LIFS937-08|Acanthuridae|BOLD:AAB0201
Pygoplites diacanthus|LIFSA196-08|Pomacanthidae|BOLD:AAC3194
Pygoplites diacanthus|LIFS300-08|Pomacanthidae|BOLD:AAC3194
Centropyge bispinosa|LIFSA209-08|Pomacanthidae|BOLD:AAB7091
Centropyge bispinosa|LIFSA223-08|Pomacanthidae|BOLD:AAB7091
Centropyge bispinosa|LIFS801-08|Pomacanthidae|BOLD:AAB7091
Centropyge bispinosa|LIFS802-08|Pomacanthidae|BOLD:AAB7091
Pomacanthus sexstriatus|LIFS299-08|Pomacanthidae|BOLD:AAE4274
Pomacanthus sexstriatus|LIFS605-08|Pomacanthidae|BOLD:AAE4274
Centropyge vrolikii|LIFSA195-08|Pomacanthidae|BOLD:AAD2580
Centropyge bicolor|LIFSA210-08|Pomacanthidae|BOLD:AAC0137
Centropyge bicolor|LIFS375-08|Pomacanthidae|BOLD:AAC0137
Centropyge bicolor|LIFS547-08|Pomacanthidae|BOLD:AAC0137
Centropyge bicolor|LIFS318-08|Pomacanthidae|BOLD:AAC0137
Lutjanus bohar|LIFS774-08|Lutjanidae|BOLD:AAB4501
Lutjanus vitta|LIFS479-08|Lutjanidae|BOLD:AAB5459
Lutjanus monostigma|LIFSA169-08|Lutjanidae|BOLD:AAB2907
Lutjanus ehrenbergii|LIFS664-08|Lutjanidae|BOLD:AAB2906
Lutjanus russellii|LIFS495-08|Lutjanidae|BOLD:AAB2904
Lutjanus russellii|LIFS496-08|Lutjanidae|BOLD:AAB2904
Lutjanus fulviflamma|LIFS187-08|Lutjanidae|BOLD:AAA9576
Lutjanus fulviflamma|LIFS297-08|Lutjanidae|BOLD:AAA9576
Lutjanus fulviflamma|LIFS497-08|Lutjanidae|BOLD:AAA9576
Lutjanus carponotatus|LIFS198-08|Lutjanidae|BOLD:AAB9713
Lutjanus carponotatus|LIFS738-08|Lutjanidae|BOLD:AAB9713
Lutjanus carponotatus|LIFS739-08|Lutjanidae|BOLD:AAB9713
Lutjanus argentimaculatus|LIFS611-08|Lutjanidae|BOLD:AAB2440
Macolor niger|LIFS770-08|Lutjanidae|BOLD:AAD1796
Caesio caerulaurea|LIFS614-08|Caesionidae|BOLD:ABZ2197
Caesio cuning|LIFS226-08|Caesionidae|BOLD:AAD1988
Caesio cuning|LIFS227-08|Caesionidae|BOLD:AAD1988
Caesio cuning|LIFS229-08|Caesionidae|BOLD:AAD1988
Caesio cuning|LIFS225-08|Caesionidae|BOLD:AAD1988
Caesio cuning|LIFS228-08|Caesionidae|BOLD:AAD1988
Siganus punctatissimus|LIFSA269-08|Siganidae|BOLD:AAJ6675
Siganus punctatus|LIFS764-08|Siganidae|BOLD:AAB2341
Siganus vulpinus|LIFS741-08|Siganidae|BOLD:AAD9299
Siganus doliatus|LIFS302-08|Siganidae|BOLD:AAC8755
Siganus corallinus|LIFSA103-08|Siganidae|BOLD:AAC8755
Siganus corallinus|LIFS765-08|Siganidae|BOLD:AAC8755
Siganus lineatus|LIFS418-08|Siganidae|BOLD:AAB2342
Siganus lineatus|LIFS416-08|Siganidae|BOLD:AAB2342
Siganus lineatus|LIFS417-08|Siganidae|BOLD:AAB2342
Naso annulatus|LIFSA054-08|Acanthuridae|BOLD:AAD4666
Naso lituratus|LIFSA188-08|Acanthuridae|BOLD:AAA8635
Naso lituratus|LIFSA254-08|Acanthuridae|BOLD:AAA8635
Naso lituratus|LIFS811-08|Acanthuridae|BOLD:AAA8635
Naso unicornis|LIFS810-08|Acanthuridae|BOLD:AAC8042
Naso brevirostris|LIFS841-08|Acanthuridae|BOLD:AAC1635
Naso brevirostris|LIFSA089-08|Acanthuridae|BOLD:AAC1635
Naso brevirostris|LIFS463-08|Acanthuridae|BOLD:AAC1635
Naso vlamingii|LIFS842-08|Acanthuridae|BOLD:AAE3624
Naso vlamingii|LIFS812-08|Acanthuridae|BOLD:AAE3624
Lethrinus harak|LIFS218-08|Lethrinidae|BOLD:AAC1520
Lethrinus harak|LIFS517-08|Lethrinidae|BOLD:AAC1520
Lethrinus atkinsoni|LIFS223-08|Lethrinidae|BOLD:ABY5451
Lethrinus atkinsoni|LIFSA075-08|Lethrinidae|BOLD:ABY5451
Lethrinus atkinsoni|LIFSA074-08|Lethrinidae|BOLD:ABY5451
Lethrinus atkinsoni|LIFS498-08|Lethrinidae|BOLD:ABY5451
Lethrinus obsoletus|LIFS221-08|Lethrinidae|BOLD:AAC1522
Lethrinus obsoletus|LIFS868-08|Lethrinidae|BOLD:AAC1522
Lethrinus obsoletus|LIFS843-08|Lethrinidae|BOLD:AAC1522
Lethrinus obsoletus|LIFS844-08|Lethrinidae|BOLD:AAC1522
Lethrinus obsoletus|LIFS231-08|Lethrinidae|BOLD:AAC1522
Lethrinus obsoletus|LIFS850-08|Lethrinidae|BOLD:AAC1522
Lethrinus xanthochilus|LIFSA071-08|Lethrinidae|BOLD:AAC1546
Lethrinus semicinctus|LIFS422-08|Lethrinidae|BOLD:AAC7350
Lethrinus semicinctus|LIFS421-08|Lethrinidae|BOLD:AAC7350
Lethrinus ravus|LIFS424-08|Lethrinidae|BOLD:AAC7349
Lethrinus ravus|LIFS851-08|Lethrinidae|BOLD:AAC7349
Pseudocheilinus hexataenia|LIFSA236-08|Labridae|BOLD:AAB7571
Pseudocheilinus hexataenia|LIFSA237-08|Labridae|BOLD:AAB7571
Pseudocheilinus hexataenia|LIFSA235-08|Labridae|BOLD:AAB7571
Priacanthus hamrur|LIFS645-08|Priacanthidae|BOLD:AAB1643

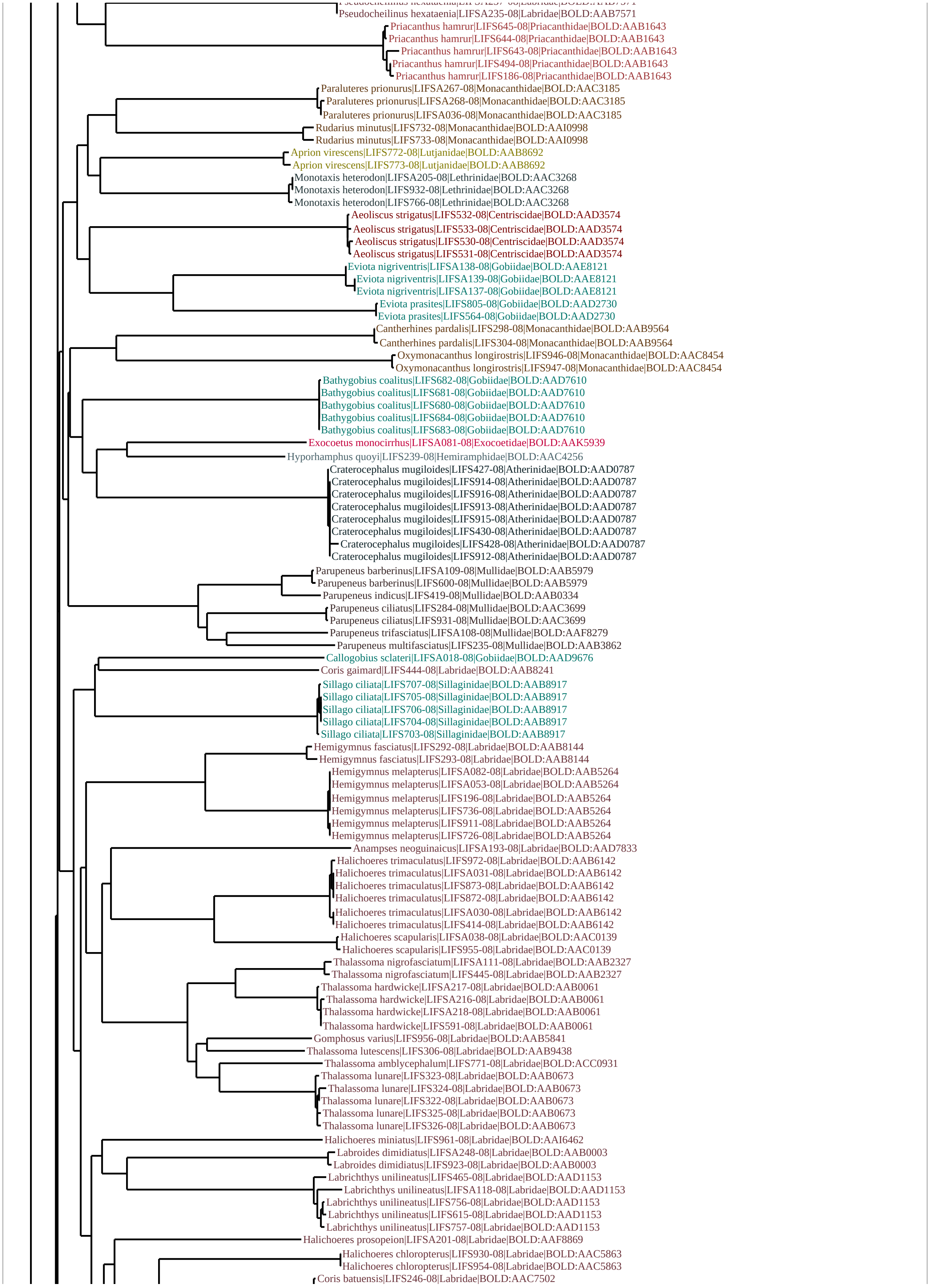
Pseudocheilinus hexataenia|LIFSA235-08|Labridae|BOLD:AAB7571
Priacanthus hamrur|LIFS645-08|Priacanthidae|BOLD:AAB1643
Priacanthus hamrur|LIFS644-08|Priacanthidae|BOLD:AAB1643
Priacanthus hamrur|LIFS643-08|Priacanthidae|BOLD:AAB1643
Priacanthus hamrur|LIFS494-08|Priacanthidae|BOLD:AAB1643
Priacanthus hamrur|LIFS186-08|Priacanthidae|BOLD:AAB1643
Paraluteres prionurus|LIFSA267-08|Monacanthidae|BOLD:AAC3185
Paraluteres prionurus|LIFSA268-08|Monacanthidae|BOLD:AAC3185
Paraluteres prionurus|LIFSA036-08|Monacanthidae|BOLD:AAC3185
Rudarius minutus|LIFS732-08|Monacanthidae|BOLD:AAI0998
Rudarius minutus|LIFS733-08|Monacanthidae|BOLD:AAI0998
Aprion virescens|LIFS772-08|Lutjanidae|BOLD:AAB8692
Aprion virescens|LIFS773-08|Lutjanidae|BOLD:AAB8692
Monotaxis heterodon|LIFSA205-08|Lethrinidae|BOLD:AAC3268
Monotaxis heterodon|LIFS932-08|Lethrinidae|BOLD:AAC3268
Monotaxis heterodon|LIFS766-08|Lethrinidae|BOLD:AAC3268
Aeoliscus strigatus|LIFS532-08|Centriscidae|BOLD:AAD3574
Aeoliscus strigatus|LIFS533-08|Centriscidae|BOLD:AAD3574
Aeoliscus strigatus|LIFS530-08|Centriscidae|BOLD:AAD3574
Aeoliscus strigatus|LIFS531-08|Centriscidae|BOLD:AAD3574
Eviota nigriventris|LIFSA138-08|Gobiidae|BOLD:AAE8121
Eviota nigriventris|LIFSA139-08|Gobiidae|BOLD:AAE8121
Eviota nigriventris|LIFSA137-08|Gobiidae|BOLD:AAE8121
Eviota prasites|LIFS805-08|Gobiidae|BOLD:AAD2730
Eviota prasites|LIFS564-08|Gobiidae|BOLD:AAD2730
Cantherhines pardalis|LIFS298-08|Monacanthidae|BOLD:AAB9564
Cantherhines pardalis|LIFS304-08|Monacanthidae|BOLD:AAB9564
Oxymonacanthus longirostris|LIFS946-08|Monacanthidae|BOLD:AAC8454
Oxymonacanthus longirostris|LIFS947-08|Monacanthidae|BOLD:AAC8454
Bathygobius coalitus|LIFS682-08|Gobiidae|BOLD:AAD7610
Bathygobius coalitus|LIFS681-08|Gobiidae|BOLD:AAD7610
Bathygobius coalitus|LIFS680-08|Gobiidae|BOLD:AAD7610
Bathygobius coalitus|LIFS684-08|Gobiidae|BOLD:AAD7610
Bathygobius coalitus|LIFS683-08|Gobiidae|BOLD:AAD7610
Exocoetus monocirrhus|LIFSA081-08|Exocoetidae|BOLD:AAK5939
Hyporhamphus quoyi|LIFS239-08|Hemiramphidae|BOLD:AAC4256
Craterocephalus mugiloides|LIFS427-08|Atherinidae|BOLD:AAD0787
Craterocephalus mugiloides|LIFS914-08|Atherinidae|BOLD:AAD0787
Craterocephalus mugiloides|LIFS916-08|Atherinidae|BOLD:AAD0787
Craterocephalus mugiloides|LIFS913-08|Atherinidae|BOLD:AAD0787
Craterocephalus mugiloides|LIFS915-08|Atherinidae|BOLD:AAD0787
Craterocephalus mugiloides|LIFS430-08|Atherinidae|BOLD:AAD0787
Craterocephalus mugiloides|LIFS428-08|Atherinidae|BOLD:AAD0787
Craterocephalus mugiloides|LIFS912-08|Atherinidae|BOLD:AAD0787
Parupeneus barberinus|LIFSA109-08|Mullidae|BOLD:AAB5979
Parupeneus barberinus|LIFS600-08|Mullidae|BOLD:AAB5979
Parupeneus indicus|LIFS419-08|Mullidae|BOLD:AAB0334
Parupeneus ciliatus|LIFS284-08|Mullidae|BOLD:AAC3699
Parupeneus ciliatus|LIFS931-08|Mullidae|BOLD:AAC3699
Parupeneus trifasciatus|LIFSA108-08|Mullidae|BOLD:AAF8279
Parupeneus multifasciatus|LIFS235-08|Mullidae|BOLD:AAB3862
Callogobius sclateri|LIFSA018-08|Gobiidae|BOLD:AAD9676
Coris gaimard|LIFS444-08|Labridae|BOLD:AAB8241
Sillago ciliata|LIFS707-08|Sillaginidae|BOLD:AAB8917
Sillago ciliata|LIFS705-08|Sillaginidae|BOLD:AAB8917
Sillago ciliata|LIFS706-08|Sillaginidae|BOLD:AAB8917
Sillago ciliata|LIFS704-08|Sillaginidae|BOLD:AAB8917
Sillago ciliata|LIFS703-08|Sillaginidae|BOLD:AAB8917
Hemigymnus fasciatus|LIFS292-08|Labridae|BOLD:AAB8144
Hemigymnus fasciatus|LIFS293-08|Labridae|BOLD:AAB8144
Hemigymnus melapterus|LIFSA082-08|Labridae|BOLD:AAB5264
Hemigymnus melapterus|LIFSA053-08|Labridae|BOLD:AAB5264
Hemigymnus melapterus|LIFS196-08|Labridae|BOLD:AAB5264
Hemigymnus melapterus|LIFS736-08|Labridae|BOLD:AAB5264
Hemigymnus melapterus|LIFS911-08|Labridae|BOLD:AAB5264
Hemigymnus melapterus|LIFS726-08|Labridae|BOLD:AAB5264
Anampses neoguinaicus|LIFSA193-08|Labridae|BOLD:AAD7833
Halichoeres trimaculatus|LIFS972-08|Labridae|BOLD:AAB6142
Halichoeres trimaculatus|LIFSA031-08|Labridae|BOLD:AAB6142
Halichoeres trimaculatus|LIFS873-08|Labridae|BOLD:AAB6142
Halichoeres trimaculatus|LIFS872-08|Labridae|BOLD:AAB6142
Halichoeres trimaculatus|LIFSA030-08|Labridae|BOLD:AAB6142
Halichoeres trimaculatus|LIFS414-08|Labridae|BOLD:AAB6142
Halichoeres scapularis|LIFSA038-08|Labridae|BOLD:AAC0139
Halichoeres scapularis|LIFS955-08|Labridae|BOLD:AAC0139
Thalassoma nigrofasciatum|LIFSA111-08|Labridae|BOLD:AAB2327
Thalassoma nigrofasciatum|LIFS445-08|Labridae|BOLD:AAB2327
Thalassoma hardwicke|LIFSA217-08|Labridae|BOLD:AAB0061
Thalassoma hardwicke|LIFSA216-08|Labridae|BOLD:AAB0061
Thalassoma hardwicke|LIFSA218-08|Labridae|BOLD:AAB0061
Thalassoma hardwicke|LIFS591-08|Labridae|BOLD:AAB0061
Gomphosus varius|LIFS956-08|Labridae|BOLD:AAB5841
Thalassoma lutescens|LIFS306-08|Labridae|BOLD:AAB9438
Thalassoma amblycephalum|LIFS771-08|Labridae|BOLD:ACC0931
Thalassoma lunare|LIFS323-08|Labridae|BOLD:AAB0673
Thalassoma lunare|LIFS324-08|Labridae|BOLD:AAB0673
Thalassoma lunare|LIFS322-08|Labridae|BOLD:AAB0673
Thalassoma lunare|LIFS325-08|Labridae|BOLD:AAB0673
Thalassoma lunare|LIFS326-08|Labridae|BOLD:AAB0673
Halichoeres miniatus|LIFS961-08|Labridae|BOLD:AAI6462
Labroides dimidiatus|LIFSA248-08|Labridae|BOLD:AAB0003
Labroides dimidiatus|LIFS923-08|Labridae|BOLD:AAB0003
Labrichthys unilineatus|LIFS465-08|Labridae|BOLD:AAD1153
Labrichthys unilineatus|LIFSA118-08|Labridae|BOLD:AAD1153
Labrichthys unilineatus|LIFS756-08|Labridae|BOLD:AAD1153
Labrichthys unilineatus|LIFS615-08|Labridae|BOLD:AAD1153
Labrichthys unilineatus|LIFS757-08|Labridae|BOLD:AAD1153
Halichoeres prosopeion|LIFSA201-08|Labridae|BOLD:AAF8869
Halichoeres chloropterus|LIFS930-08|Labridae|BOLD:AAC5863
Halichoeres chloropterus|LIFS954-08|Labridae|BOLD:AAC5863
Coris batuensis|LIFS246-08|Labridae|BOLD:AAC7502

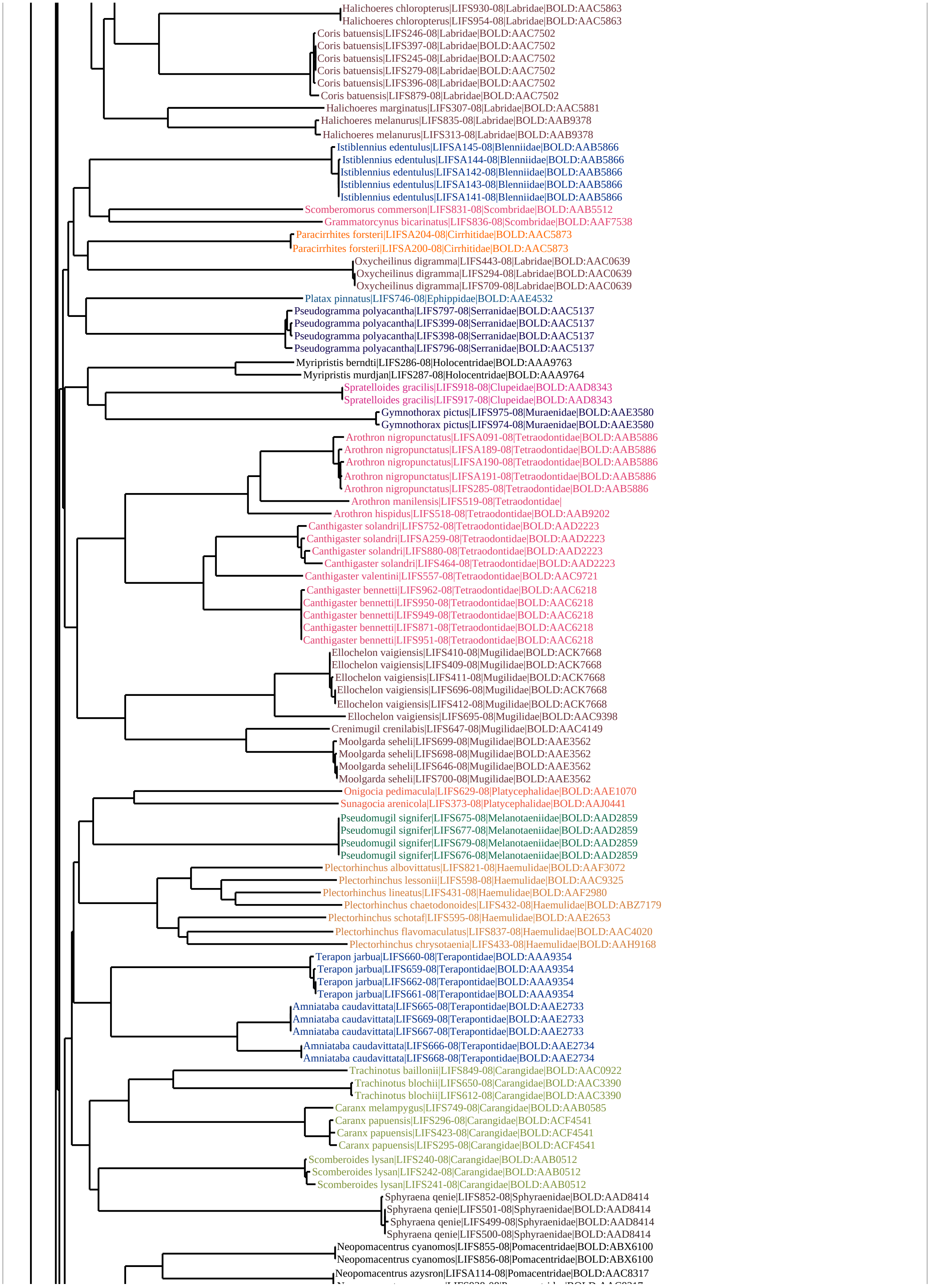
Halichoeres chloropterus|LIFS930-08|Labridae|BOLD:AAC5863
Halichoeres chloropterus|LIFS954-08|Labridae|BOLD:AAC5863
Coris batuensis|LIFS246-08|Labridae|BOLD:AAC7502
Coris batuensis|LIFS397-08|Labridae|BOLD:AAC7502
Coris batuensis|LIFS245-08|Labridae|BOLD:AAC7502
Coris batuensis|LIFS279-08|Labridae|BOLD:AAC7502
Coris batuensis|LIFS396-08|Labridae|BOLD:AAC7502
Coris batuensis|LIFS879-08|Labridae|BOLD:AAC7502
Halichoeres marginatus|LIFS307-08|Labridae|BOLD:AAC5881
Halichoeres melanurus|LIFS835-08|Labridae|BOLD:AAB9378
Halichoeres melanurus|LIFS313-08|Labridae|BOLD:AAB9378
Istiblennius edentulus|LIFSA145-08|Blenniidae|BOLD:AAB5866
Istiblennius edentulus|LIFSA144-08|Blenniidae|BOLD:AAB5866
Istiblennius edentulus|LIFSA142-08|Blenniidae|BOLD:AAB5866
Istiblennius edentulus|LIFSA143-08|Blenniidae|BOLD:AAB5866
Istiblennius edentulus|LIFSA141-08|Blenniidae|BOLD:AAB5866
Scomberomorus commerson|LIFS831-08|Scombridae|BOLD:AAB5512
Grammatorcynus bicarinatus|LIFS836-08|Scombridae|BOLD:AAF7538
Paracirrhites forsteri|LIFSA204-08|Cirrhitidae|BOLD:AAC5873
Paracirrhites forsteri|LIFSA200-08|Cirrhitidae|BOLD:AAC5873
Oxycheilinus digramma|LIFS443-08|Labridae|BOLD:AAC0639
Oxycheilinus digramma|LIFS294-08|Labridae|BOLD:AAC0639
Oxycheilinus digramma|LIFS709-08|Labridae|BOLD:AAC0639
Platax pinnatus|LIFS746-08|Ephippidae|BOLD:AAE4532
Pseudogramma polyacantha|LIFS797-08|Serranidae|BOLD:AAC5137
Pseudogramma polyacantha|LIFS399-08|Serranidae|BOLD:AAC5137
Pseudogramma polyacantha|LIFS398-08|Serranidae|BOLD:AAC5137
Pseudogramma polyacantha|LIFS796-08|Serranidae|BOLD:AAC5137
Myripristis berndti|LIFS286-08|Holocentridae|BOLD:AAA9763
Myripristis murdjan|LIFS287-08|Holocentridae|BOLD:AAA9764
Spratelloides gracilis|LIFS918-08|Clupeidae|BOLD:AAD8343
Spratelloides gracilis|LIFS917-08|Clupeidae|BOLD:AAD8343
Gymnothorax pictus|LIFS975-08|Muraenidae|BOLD:AAE3580
Gymnothorax pictus|LIFS974-08|Muraenidae|BOLD:AAE3580
Arothron nigropunctatus|LIFSA091-08|Tetraodontidae|BOLD:AAB5886
Arothron nigropunctatus|LIFSA189-08|Tetraodontidae|BOLD:AAB5886
Arothron nigropunctatus|LIFSA190-08|Tetraodontidae|BOLD:AAB5886
Arothron nigropunctatus|LIFSA191-08|Tetraodontidae|BOLD:AAB5886
Arothron nigropunctatus|LIFS285-08|Tetraodontidae|BOLD:AAB5886
Arothron manilensis|LIFS519-08|Tetraodontidae|
Arothron hispidus|LIFS518-08|Tetraodontidae|BOLD:AAB9202
Canthigaster solandri|LIFS752-08|Tetraodontidae|BOLD:AAD2223
Canthigaster solandri|LIFSA259-08|Tetraodontidae|BOLD:AAD2223
Canthigaster solandri|LIFS880-08|Tetraodontidae|BOLD:AAD2223
Canthigaster solandri|LIFS464-08|Tetraodontidae|BOLD:AAD2223
Canthigaster valentini|LIFS557-08|Tetraodontidae|BOLD:AAC9721
Canthigaster bennetti|LIFS962-08|Tetraodontidae|BOLD:AAC6218
Canthigaster bennetti|LIFS950-08|Tetraodontidae|BOLD:AAC6218
Canthigaster bennetti|LIFS949-08|Tetraodontidae|BOLD:AAC6218
Canthigaster bennetti|LIFS871-08|Tetraodontidae|BOLD:AAC6218
Canthigaster bennetti|LIFS951-08|Tetraodontidae|BOLD:AAC6218
Ellochelon vaigiensis|LIFS410-08|Mugilidae|BOLD:ACK7668
Ellochelon vaigiensis|LIFS409-08|Mugilidae|BOLD:ACK7668
Ellochelon vaigiensis|LIFS411-08|Mugilidae|BOLD:ACK7668
Ellochelon vaigiensis|LIFS696-08|Mugilidae|BOLD:ACK7668
Ellochelon vaigiensis|LIFS412-08|Mugilidae|BOLD:ACK7668
Ellochelon vaigiensis|LIFS695-08|Mugilidae|BOLD:AAC9398
Crenimugil crenilabis|LIFS647-08|Mugilidae|BOLD:AAC4149
Moolgarda seheli|LIFS699-08|Mugilidae|BOLD:AAE3562
Moolgarda seheli|LIFS698-08|Mugilidae|BOLD:AAE3562
Moolgarda seheli|LIFS646-08|Mugilidae|BOLD:AAE3562
Moolgarda seheli|LIFS700-08|Mugilidae|BOLD:AAE3562
Onigocia pedimacula|LIFS629-08|Platycephalidae|BOLD:AAE1070
Sunagocia arenicola|LIFS373-08|Platycephalidae|BOLD:AAJ0441
Pseudomugil signifer|LIFS675-08|Melanotaeniidae|BOLD:AAD2859
Pseudomugil signifer|LIFS677-08|Melanotaeniidae|BOLD:AAD2859
Pseudomugil signifer|LIFS679-08|Melanotaeniidae|BOLD:AAD2859
Pseudomugil signifer|LIFS676-08|Melanotaeniidae|BOLD:AAD2859
Plectorhinchus albovittatus|LIFS821-08|Haemulidae|BOLD:AAF3072
Plectorhinchus lessonii|LIFS598-08|Haemulidae|BOLD:AAC9325
Plectorhinchus lineatus|LIFS431-08|Haemulidae|BOLD:AAF2980
Plectorhinchus chaetodonoides|LIFS432-08|Haemulidae|BOLD:ABZ7179
Plectorhinchus schotaf|LIFS595-08|Haemulidae|BOLD:AAE2653
Plectorhinchus flavomaculatus|LIFS837-08|Haemulidae|BOLD:AAC4020
Plectorhinchus chrysotaenia|LIFS433-08|Haemulidae|BOLD:AAH9168
Terapon jarbua|LIFS660-08|Terapontidae|BOLD:AAA9354
Terapon jarbua|LIFS659-08|Terapontidae|BOLD:AAA9354
Terapon jarbua|LIFS662-08|Terapontidae|BOLD:AAA9354
Terapon jarbua|LIFS661-08|Terapontidae|BOLD:AAA9354
Amniataba caudavittata|LIFS665-08|Terapontidae|BOLD:AAE2733
Amniataba caudavittata|LIFS669-08|Terapontidae|BOLD:AAE2733
Amniataba caudavittata|LIFS667-08|Terapontidae|BOLD:AAE2733
Amniataba caudavittata|LIFS666-08|Terapontidae|BOLD:AAE2734
Amniataba caudavittata|LIFS668-08|Terapontidae|BOLD:AAE2734
Trachinotus baillonii|LIFS849-08|Carangidae|BOLD:AAC0922
Trachinotus blochii|LIFS650-08|Carangidae|BOLD:AAC3390
Trachinotus blochii|LIFS612-08|Carangidae|BOLD:AAC3390
Caranx melampygus|LIFS749-08|Carangidae|BOLD:AAB0585
Caranx papuensis|LIFS296-08|Carangidae|BOLD:ACF4541
Caranx papuensis|LIFS423-08|Carangidae|BOLD:ACF4541
Caranx papuensis|LIFS295-08|Carangidae|BOLD:ACF4541
Scomberoides lysan|LIFS240-08|Carangidae|BOLD:AAB0512
Scomberoides lysan|LIFS242-08|Carangidae|BOLD:AAB0512
Scomberoides lysan|LIFS241-08|Carangidae|BOLD:AAB0512
Sphyraena qenie|LIFS852-08|Sphyraenidae|BOLD:AAD8414
Sphyraena qenie|LIFS501-08|Sphyraenidae|BOLD:AAD8414
Sphyraena qenie|LIFS499-08|Sphyraenidae|BOLD:AAD8414
Sphyraena qenie|LIFS500-08|Sphyraenidae|BOLD:AAD8414
Neopomacentrus cyanomos|LIFS855-08|Pomacentridae|BOLD:ABX6100
Neopomacentrus cyanomos|LIFS856-08|Pomacentridae|BOLD:ABX6100
Neopomacentrus azysron|LIFSA114-08|Pomacentridae|BOLD:AAC8317

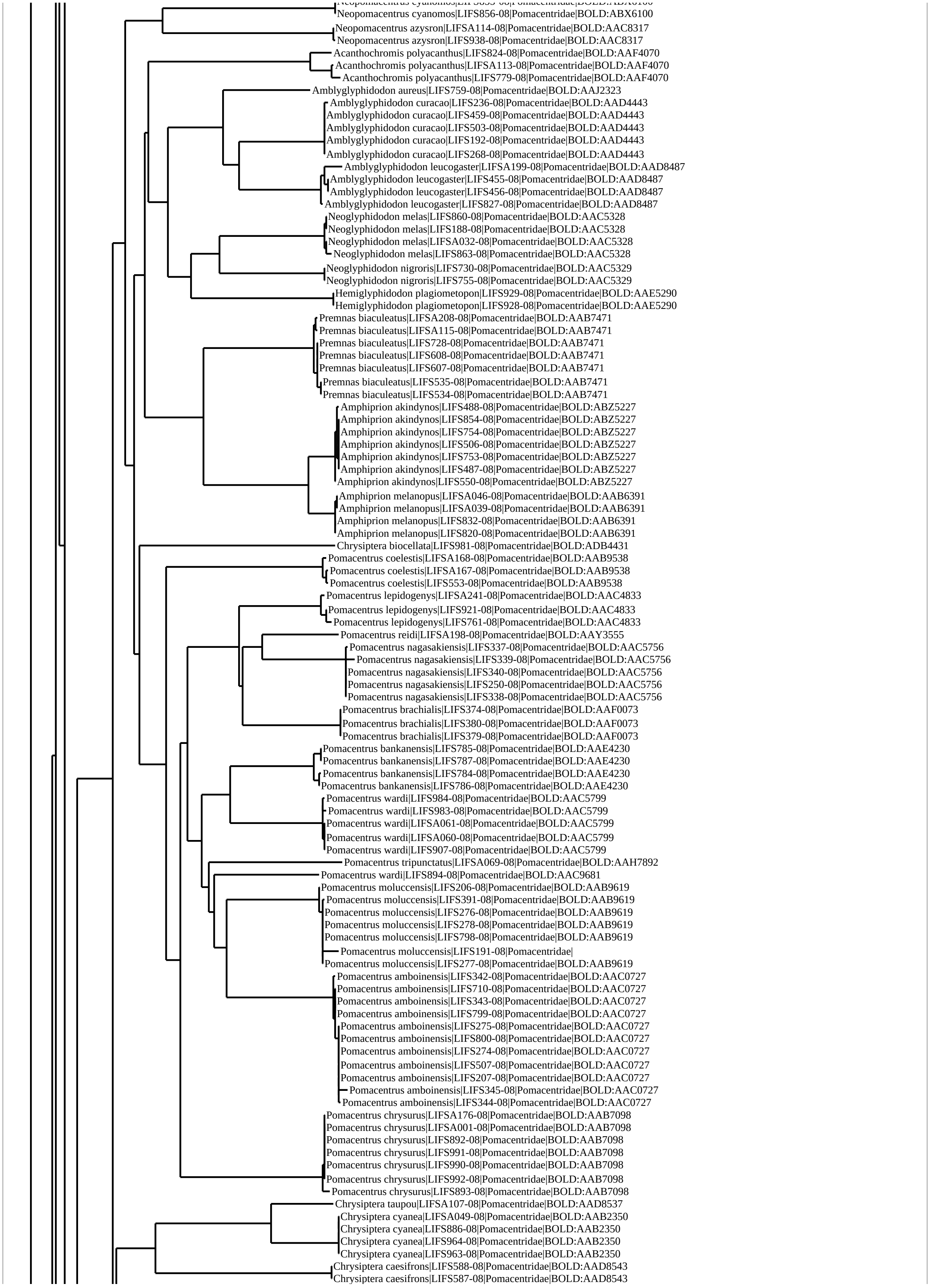
Neopomacentrus cyanomos|LIFS856-08|Pomacentridae|BOLD:ABX6100
Neopomacentrus azysron|LIFSA114-08|Pomacentridae|BOLD:AAC8317
Neopomacentrus azysron|LIFS938-08|Pomacentridae|BOLD:AAC8317
Acanthochromis polyacanthus|LIFS824-08|Pomacentridae|BOLD:AAF4070
Acanthochromis polyacanthus|LIFSA113-08|Pomacentridae|BOLD:AAF4070
Acanthochromis polyacanthus|LIFS779-08|Pomacentridae|BOLD:AAF4070
Amblyglyphidodon aureus|LIFS759-08|Pomacentridae|BOLD:AAJ2323
Amblyglyphidodon curacao|LIFS236-08|Pomacentridae|BOLD:AAD4443
Amblyglyphidodon curacao|LIFS459-08|Pomacentridae|BOLD:AAD4443
Amblyglyphidodon curacao|LIFS503-08|Pomacentridae|BOLD:AAD4443
Amblyglyphidodon curacao|LIFS192-08|Pomacentridae|BOLD:AAD4443
Amblyglyphidodon curacao|LIFS268-08|Pomacentridae|BOLD:AAD4443
Amblyglyphidodon leucogaster|LIFSA199-08|Pomacentridae|BOLD:AAD8487
Amblyglyphidodon leucogaster|LIFS455-08|Pomacentridae|BOLD:AAD8487
Amblyglyphidodon leucogaster|LIFS456-08|Pomacentridae|BOLD:AAD8487
Amblyglyphidodon leucogaster|LIFS827-08|Pomacentridae|BOLD:AAD8487
Neoglyphidodon melas|LIFS860-08|Pomacentridae|BOLD:AAC5328
Neoglyphidodon melas|LIFS188-08|Pomacentridae|BOLD:AAC5328
Neoglyphidodon melas|LIFSA032-08|Pomacentridae|BOLD:AAC5328
Neoglyphidodon melas|LIFS863-08|Pomacentridae|BOLD:AAC5328
Neoglyphidodon nigroris|LIFS730-08|Pomacentridae|BOLD:AAC5329
Neoglyphidodon nigroris|LIFS755-08|Pomacentridae|BOLD:AAC5329
Hemiglyphidodon plagiometopon|LIFS929-08|Pomacentridae|BOLD:AAE5290
Hemiglyphidodon plagiometopon|LIFS928-08|Pomacentridae|BOLD:AAE5290
Premnas biaculeatus|LIFSA208-08|Pomacentridae|BOLD:AAB7471
Premnas biaculeatus|LIFSA115-08|Pomacentridae|BOLD:AAB7471
Premnas biaculeatus|LIFS728-08|Pomacentridae|BOLD:AAB7471
Premnas biaculeatus|LIFS608-08|Pomacentridae|BOLD:AAB7471
Premnas biaculeatus|LIFS607-08|Pomacentridae|BOLD:AAB7471
Premnas biaculeatus|LIFS535-08|Pomacentridae|BOLD:AAB7471
Premnas biaculeatus|LIFS534-08|Pomacentridae|BOLD:AAB7471
Amphiprion akindynos|LIFS488-08|Pomacentridae|BOLD:ABZ5227
Amphiprion akindynos|LIFS854-08|Pomacentridae|BOLD:ABZ5227
Amphiprion akindynos|LIFS754-08|Pomacentridae|BOLD:ABZ5227
Amphiprion akindynos|LIFS506-08|Pomacentridae|BOLD:ABZ5227
Amphiprion akindynos|LIFS753-08|Pomacentridae|BOLD:ABZ5227
Amphiprion akindynos|LIFS487-08|Pomacentridae|BOLD:ABZ5227
Amphiprion akindynos|LIFS550-08|Pomacentridae|BOLD:ABZ5227
Amphiprion melanopus|LIFSA046-08|Pomacentridae|BOLD:AAB6391
Amphiprion melanopus|LIFSA039-08|Pomacentridae|BOLD:AAB6391
Amphiprion melanopus|LIFS832-08|Pomacentridae|BOLD:AAB6391
Amphiprion melanopus|LIFS820-08|Pomacentridae|BOLD:AAB6391
Chrysiptera biocellata|LIFS981-08|Pomacentridae|BOLD:ADB4431
Pomacentrus coelestis|LIFSA168-08|Pomacentridae|BOLD:AAB9538
Pomacentrus coelestis|LIFSA167-08|Pomacentridae|BOLD:AAB9538
Pomacentrus coelestis|LIFS553-08|Pomacentridae|BOLD:AAB9538
Pomacentrus lepidogenys|LIFSA241-08|Pomacentridae|BOLD:AAC4833
Pomacentrus lepidogenys|LIFS921-08|Pomacentridae|BOLD:AAC4833
Pomacentrus lepidogenys|LIFS761-08|Pomacentridae|BOLD:AAC4833
Pomacentrus reidi|LIFSA198-08|Pomacentridae|BOLD:AAY3555
Pomacentrus nagasakiensis|LIFS337-08|Pomacentridae|BOLD:AAC5756
Pomacentrus nagasakiensis|LIFS339-08|Pomacentridae|BOLD:AAC5756
Pomacentrus nagasakiensis|LIFS340-08|Pomacentridae|BOLD:AAC5756
Pomacentrus nagasakiensis|LIFS250-08|Pomacentridae|BOLD:AAC5756
Pomacentrus nagasakiensis|LIFS338-08|Pomacentridae|BOLD:AAC5756
Pomacentrus brachialis|LIFS374-08|Pomacentridae|BOLD:AAF0073
Pomacentrus brachialis|LIFS380-08|Pomacentridae|BOLD:AAF0073
Pomacentrus brachialis|LIFS379-08|Pomacentridae|BOLD:AAF0073
Pomacentrus bankanensis|LIFS785-08|Pomacentridae|BOLD:AAE4230
Pomacentrus bankanensis|LIFS787-08|Pomacentridae|BOLD:AAE4230
Pomacentrus bankanensis|LIFS784-08|Pomacentridae|BOLD:AAE4230
Pomacentrus bankanensis|LIFS786-08|Pomacentridae|BOLD:AAE4230
Pomacentrus wardi|LIFS984-08|Pomacentridae|BOLD:AAC5799
Pomacentrus wardi|LIFS983-08|Pomacentridae|BOLD:AAC5799
Pomacentrus wardi|LIFSA061-08|Pomacentridae|BOLD:AAC5799
Pomacentrus wardi|LIFSA060-08|Pomacentridae|BOLD:AAC5799
Pomacentrus wardi|LIFS907-08|Pomacentridae|BOLD:AAC5799
Pomacentrus tripunctatus|LIFSA069-08|Pomacentridae|BOLD:AAH7892
Pomacentrus wardi|LIFS894-08|Pomacentridae|BOLD:AAC9681
Pomacentrus moluccensis|LIFS206-08|Pomacentridae|BOLD:AAB9619
Pomacentrus moluccensis|LIFS391-08|Pomacentridae|BOLD:AAB9619
Pomacentrus moluccensis|LIFS276-08|Pomacentridae|BOLD:AAB9619
Pomacentrus moluccensis|LIFS278-08|Pomacentridae|BOLD:AAB9619
Pomacentrus moluccensis|LIFS798-08|Pomacentridae|BOLD:AAB9619
Pomacentrus moluccensis|LIFS191-08|Pomacentridae|
Pomacentrus moluccensis|LIFS277-08|Pomacentridae|BOLD:AAB9619
Pomacentrus amboinensis|LIFS342-08|Pomacentridae|BOLD:AAC0727
Pomacentrus amboinensis|LIFS710-08|Pomacentridae|BOLD:AAC0727
Pomacentrus amboinensis|LIFS343-08|Pomacentridae|BOLD:AAC0727
Pomacentrus amboinensis|LIFS799-08|Pomacentridae|BOLD:AAC0727
Pomacentrus amboinensis|LIFS275-08|Pomacentridae|BOLD:AAC0727
Pomacentrus amboinensis|LIFS800-08|Pomacentridae|BOLD:AAC0727
Pomacentrus amboinensis|LIFS274-08|Pomacentridae|BOLD:AAC0727
Pomacentrus amboinensis|LIFS507-08|Pomacentridae|BOLD:AAC0727
Pomacentrus amboinensis|LIFS207-08|Pomacentridae|BOLD:AAC0727
Pomacentrus amboinensis|LIFS345-08|Pomacentridae|BOLD:AAC0727
Pomacentrus amboinensis|LIFS344-08|Pomacentridae|BOLD:AAC0727
Pomacentrus chrysurus|LIFSA176-08|Pomacentridae|BOLD:AAB7098
Pomacentrus chrysurus|LIFSA001-08|Pomacentridae|BOLD:AAB7098
Pomacentrus chrysurus|LIFS892-08|Pomacentridae|BOLD:AAB7098
Pomacentrus chrysurus|LIFS991-08|Pomacentridae|BOLD:AAB7098
Pomacentrus chrysurus|LIFS990-08|Pomacentridae|BOLD:AAB7098
Pomacentrus chrysurus|LIFS992-08|Pomacentridae|BOLD:AAB7098
Pomacentrus chrysurus|LIFS893-08|Pomacentridae|BOLD:AAB7098
Chrysiptera taupou|LIFSA107-08|Pomacentridae|BOLD:AAD8537
Chrysiptera cyanea|LIFSA049-08|Pomacentridae|BOLD:AAB2350
Chrysiptera cyanea|LIFS886-08|Pomacentridae|BOLD:AAB2350
Chrysiptera cyanea|LIFS964-08|Pomacentridae|BOLD:AAB2350
Chrysiptera cyanea|LIFS963-08|Pomacentridae|BOLD:AAB2350
Chrysiptera caesifrons|LIFS588-08|Pomacentridae|BOLD:AAD8543
Chrysiptera caesifrons|LIFS587-08|Pomacentridae|BOLD:AAD8543

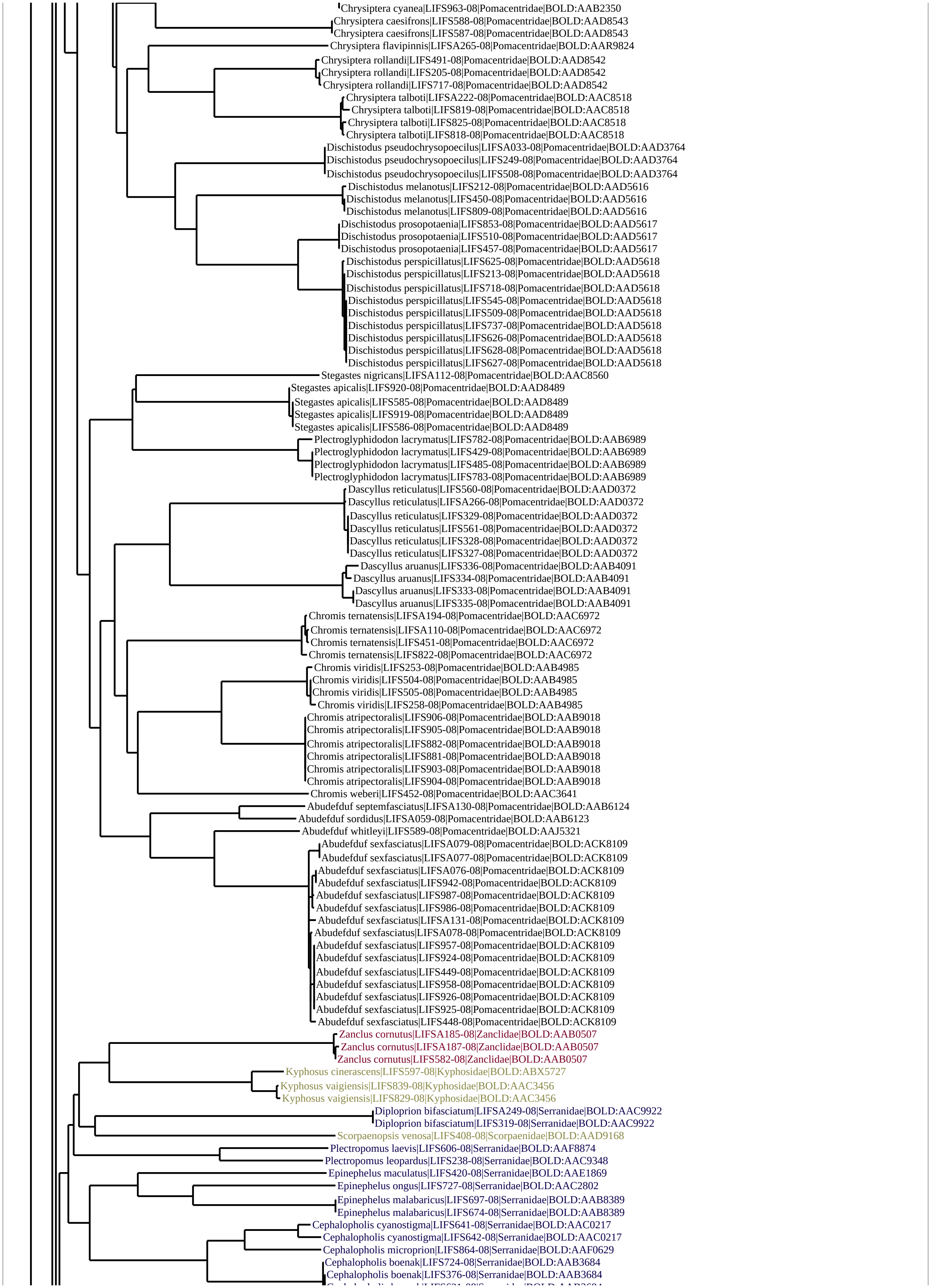

Chrysiptera cyanea|LIFS963-08|Pomacentridae|BOLD:AAB2350
Chrysiptera caesifrons|LIFS588-08|Pomacentridae|BOLD:AAD8543
Chrysiptera caesifrons|LIFS587-08|Pomacentridae|BOLD:AAD8543
Chrysiptera flavipinnis|LIFSA265-08|Pomacentridae|BOLD:AAR9824
Chrysiptera rollandi|LIFS491-08|Pomacentridae|BOLD:AAD8542
Chrysiptera rollandi|LIFS205-08|Pomacentridae|BOLD:AAD8542
Chrysiptera rollandi|LIFS717-08|Pomacentridae|BOLD:AAD8542
Chrysiptera talboti|LIFSA222-08|Pomacentridae|BOLD:AAC8518
Chrysiptera talboti|LIFS819-08|Pomacentridae|BOLD:AAC8518
Chrysiptera talboti|LIFS825-08|Pomacentridae|BOLD:AAC8518
Chrysiptera talboti|LIFS818-08|Pomacentridae|BOLD:AAC8518
Dischistodus pseudochrysopoecilus|LIFSA033-08|Pomacentridae|BOLD:AAD3764
Dischistodus pseudochrysopoecilus|LIFS249-08|Pomacentridae|BOLD:AAD3764
Dischistodus pseudochrysopoecilus|LIFS508-08|Pomacentridae|BOLD:AAD3764
Dischistodus melanotus|LIFS212-08|Pomacentridae|BOLD:AAD5616
Dischistodus melanotus|LIFS450-08|Pomacentridae|BOLD:AAD5616
Dischistodus melanotus|LIFS809-08|Pomacentridae|BOLD:AAD5616
Dischistodus prosopotaenia|LIFS853-08|Pomacentridae|BOLD:AAD5617
Dischistodus prosopotaenia|LIFS510-08|Pomacentridae|BOLD:AAD5617
Dischistodus prosopotaenia|LIFS457-08|Pomacentridae|BOLD:AAD5617
Dischistodus perspicillatus|LIFS625-08|Pomacentridae|BOLD:AAD5618
Dischistodus perspicillatus|LIFS213-08|Pomacentridae|BOLD:AAD5618
Dischistodus perspicillatus|LIFS718-08|Pomacentridae|BOLD:AAD5618
Dischistodus perspicillatus|LIFS545-08|Pomacentridae|BOLD:AAD5618
Dischistodus perspicillatus|LIFS509-08|Pomacentridae|BOLD:AAD5618
Dischistodus perspicillatus|LIFS737-08|Pomacentridae|BOLD:AAD5618
Dischistodus perspicillatus|LIFS626-08|Pomacentridae|BOLD:AAD5618
Dischistodus perspicillatus|LIFS628-08|Pomacentridae|BOLD:AAD5618
Dischistodus perspicillatus|LIFS627-08|Pomacentridae|BOLD:AAD5618
Stegastes nigricans|LIFSA112-08|Pomacentridae|BOLD:AAC8560
Stegastes apicalis|LIFS920-08|Pomacentridae|BOLD:AAD8489
Stegastes apicalis|LIFS585-08|Pomacentridae|BOLD:AAD8489
Stegastes apicalis|LIFS919-08|Pomacentridae|BOLD:AAD8489
Stegastes apicalis|LIFS586-08|Pomacentridae|BOLD:AAD8489
Plectroglyphidodon lacrymatus|LIFS782-08|Pomacentridae|BOLD:AAB6989
Plectroglyphidodon lacrymatus|LIFS429-08|Pomacentridae|BOLD:AAB6989
Plectroglyphidodon lacrymatus|LIFS485-08|Pomacentridae|BOLD:AAB6989
Plectroglyphidodon lacrymatus|LIFS783-08|Pomacentridae|BOLD:AAB6989
Dascyllus reticulatus|LIFS560-08|Pomacentridae|BOLD:AAD0372
Dascyllus reticulatus|LIFSA266-08|Pomacentridae|BOLD:AAD0372
Dascyllus reticulatus|LIFS329-08|Pomacentridae|BOLD:AAD0372
Dascyllus reticulatus|LIFS561-08|Pomacentridae|BOLD:AAD0372
Dascyllus reticulatus|LIFS328-08|Pomacentridae|BOLD:AAD0372
Dascyllus reticulatus|LIFS327-08|Pomacentridae|BOLD:AAD0372
Dascyllus aruanus|LIFS336-08|Pomacentridae|BOLD:AAB4091
Dascyllus aruanus|LIFS334-08|Pomacentridae|BOLD:AAB4091
Dascyllus aruanus|LIFS333-08|Pomacentridae|BOLD:AAB4091
Dascyllus aruanus|LIFS335-08|Pomacentridae|BOLD:AAB4091
Chromis ternatensis|LIFSA194-08|Pomacentridae|BOLD:AAC6972
Chromis ternatensis|LIFSA110-08|Pomacentridae|BOLD:AAC6972
Chromis ternatensis|LIFS451-08|Pomacentridae|BOLD:AAC6972
Chromis ternatensis|LIFS822-08|Pomacentridae|BOLD:AAC6972
Chromis viridis|LIFS253-08|Pomacentridae|BOLD:AAB4985
Chromis viridis|LIFS504-08|Pomacentridae|BOLD:AAB4985
Chromis viridis|LIFS505-08|Pomacentridae|BOLD:AAB4985
Chromis viridis|LIFS258-08|Pomacentridae|BOLD:AAB4985
Chromis atripectoralis|LIFS906-08|Pomacentridae|BOLD:AAB9018
Chromis atripectoralis|LIFS905-08|Pomacentridae|BOLD:AAB9018
Chromis atripectoralis|LIFS882-08|Pomacentridae|BOLD:AAB9018
Chromis atripectoralis|LIFS881-08|Pomacentridae|BOLD:AAB9018
Chromis atripectoralis|LIFS903-08|Pomacentridae|BOLD:AAB9018
Chromis atripectoralis|LIFS904-08|Pomacentridae|BOLD:AAB9018
Chromis weberi|LIFS452-08|Pomacentridae|BOLD:AAC3641
Abudefduf septemfasciatus|LIFSA130-08|Pomacentridae|BOLD:AAB6124
Abudefduf sordidus|LIFSA059-08|Pomacentridae|BOLD:AAB6123
Abudefduf whitleyi|LIFS589-08|Pomacentridae|BOLD:AAJ5321
Abudefduf sexfasciatus|LIFSA079-08|Pomacentridae|BOLD:ACK8109
Abudefduf sexfasciatus|LIFSA077-08|Pomacentridae|BOLD:ACK8109
Abudefduf sexfasciatus|LIFSA076-08|Pomacentridae|BOLD:ACK8109
Abudefduf sexfasciatus|LIFS942-08|Pomacentridae|BOLD:ACK8109
Abudefduf sexfasciatus|LIFS987-08|Pomacentridae|BOLD:ACK8109
Abudefduf sexfasciatus|LIFS986-08|Pomacentridae|BOLD:ACK8109
Abudefduf sexfasciatus|LIFSA131-08|Pomacentridae|BOLD:ACK8109
Abudefduf sexfasciatus|LIFSA078-08|Pomacentridae|BOLD:ACK8109
Abudefduf sexfasciatus|LIFS957-08|Pomacentridae|BOLD:ACK8109
Abudefduf sexfasciatus|LIFS924-08|Pomacentridae|BOLD:ACK8109
Abudefduf sexfasciatus|LIFS449-08|Pomacentridae|BOLD:ACK8109
Abudefduf sexfasciatus|LIFS958-08|Pomacentridae|BOLD:ACK8109
Abudefduf sexfasciatus|LIFS926-08|Pomacentridae|BOLD:ACK8109
Abudefduf sexfasciatus|LIFS925-08|Pomacentridae|BOLD:ACK8109
Abudefduf sexfasciatus|LIFS448-08|Pomacentridae|BOLD:ACK8109
Zanclus cornutus|LIFSA185-08|Zanclidae|BOLD:AAB0507
Zanclus cornutus|LIFSA187-08|Zanclidae|BOLD:AAB0507
Zanclus cornutus|LIFS582-08|Zanclidae|BOLD:AAB0507
Kyphosus cinerascens|LIFS597-08|Kyphosidae|BOLD:ABX5727
Kyphosus vaigiensis|LIFS839-08|Kyphosidae|BOLD:AAC3456
Kyphosus vaigiensis|LIFS829-08|Kyphosidae|BOLD:AAC3456
Diploprion bifasciatum|LIFSA249-08|Serranidae|BOLD:AAC9922
Diploprion bifasciatum|LIFS319-08|Serranidae|BOLD:AAC9922
Scorpaenopsis venosa|LIFS408-08|Scorpaenidae|BOLD:AAD9168
Plectropomus laevis|LIFS606-08|Serranidae|BOLD:AAF8874
Plectropomus leopardus|LIFS238-08|Serranidae|BOLD:AAC9348
Epinephelus maculatus|LIFS420-08|Serranidae|BOLD:AAE1869
Epinephelus ongus|LIFS727-08|Serranidae|BOLD:AAC2802
Epinephelus malabaricus|LIFS697-08|Serranidae|BOLD:AAB8389
Epinephelus malabaricus|LIFS674-08|Serranidae|BOLD:AAB8389
Cephalopholis cyanostigma|LIFS641-08|Serranidae|BOLD:AAC0217
Cephalopholis cyanostigma|LIFS642-08|Serranidae|BOLD:AAC0217
Cephalopholis microprion|LIFS864-08|Serranidae|BOLD:AAF0629
Cephalopholis boenak|LIFS724-08|Serranidae|BOLD:AAB3684
Cephalopholis boenak|LIFS376-08|Serranidae|BOLD:AAB3684

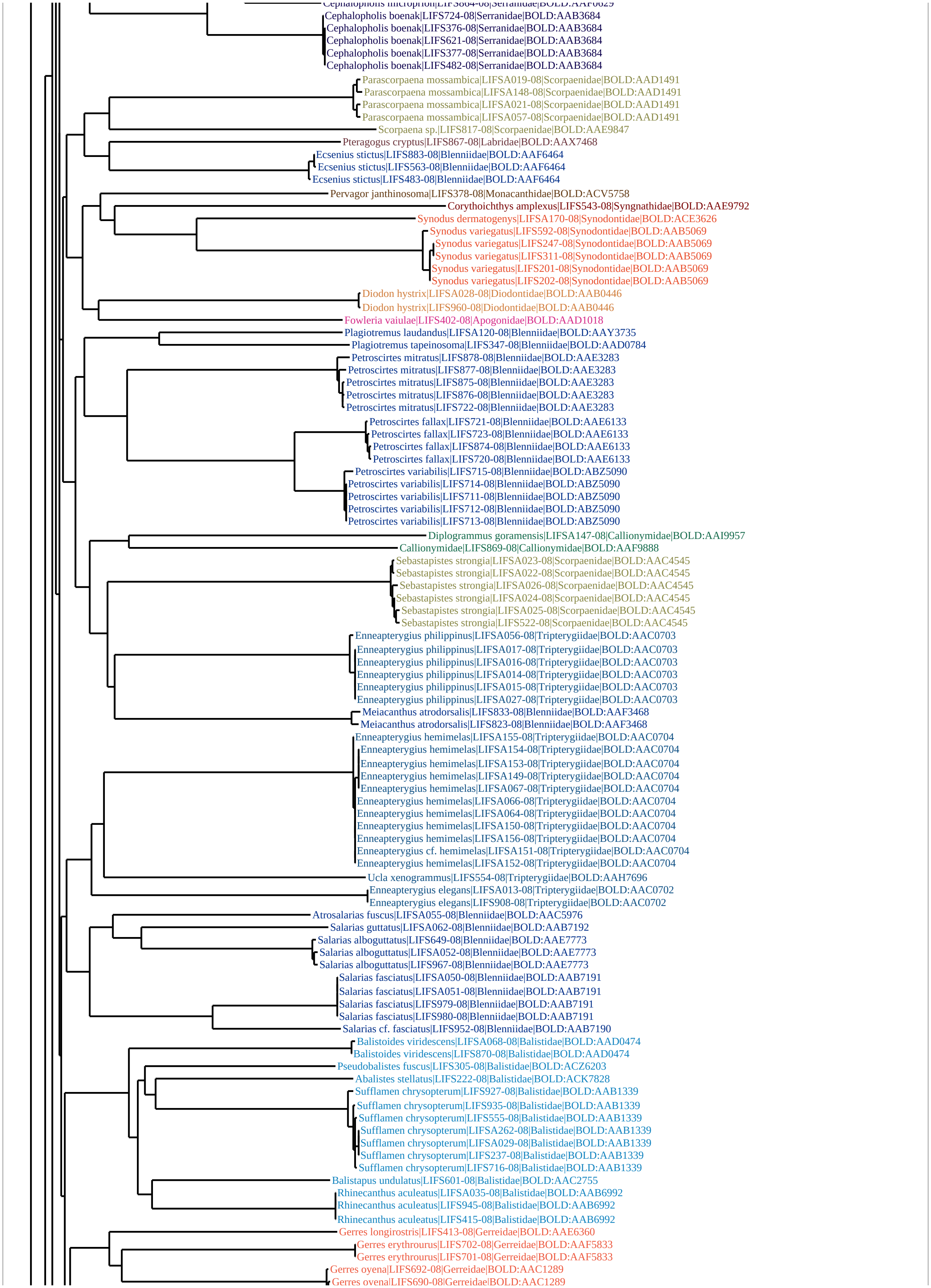
Cephalopholis boenak|LIFS724-08|Serranidae|BOLD:AAB3684
Cephalopholis boenak|LIFS376-08|Serranidae|BOLD:AAB3684
Cephalopholis boenak|LIFS621-08|Serranidae|BOLD:AAB3684
Cephalopholis boenak|LIFS377-08|Serranidae|BOLD:AAB3684
Cephalopholis boenak|LIFS482-08|Serranidae|BOLD:AAB3684
Parascorpaena mossambica|LIFSA019-08|Scorpaenidae|BOLD:AAD1491
Parascorpaena mossambica|LIFSA148-08|Scorpaenidae|BOLD:AAD1491
Parascorpaena mossambica|LIFSA021-08|Scorpaenidae|BOLD:AAD1491
Parascorpaena mossambica|LIFSA057-08|Scorpaenidae|BOLD:AAD1491
Scorpaena sp.|LIFS817-08|Scorpaenidae|BOLD:AAE9847
Pteragogus cryptus|LIFS867-08|Labridae|BOLD:AAX7468
Ecsenius stictus|LIFS883-08|Blenniidae|BOLD:AAF6464
Ecsenius stictus|LIFS563-08|Blenniidae|BOLD:AAF6464
Ecsenius stictus|LIFS483-08|Blenniidae|BOLD:AAF6464
Pervagor janthinosoma|LIFS378-08|Monacanthidae|BOLD:ACV5758
Corythoichthys amplexus|LIFS543-08|Syngnathidae|BOLD:AAE9792
Synodus dermatogenys|LIFSA170-08|Synodontidae|BOLD:ACE3626
Synodus variegatus|LIFS592-08|Synodontidae|BOLD:AAB5069
Synodus variegatus|LIFS247-08|Synodontidae|BOLD:AAB5069
Synodus variegatus|LIFS311-08|Synodontidae|BOLD:AAB5069
Synodus variegatus|LIFS201-08|Synodontidae|BOLD:AAB5069
Synodus variegatus|LIFS202-08|Synodontidae|BOLD:AAB5069
Diodon hystrix|LIFSA028-08|Diodontidae|BOLD:AAB0446
Diodon hystrix|LIFS960-08|Diodontidae|BOLD:AAB0446
Fowleria vaiulae|LIFS402-08|Apogonidae|BOLD:AAD1018
Plagiotremus laudandus|LIFSA120-08|Blenniidae|BOLD:AAY3735
Plagiotremus tapeinosoma|LIFS347-08|Blenniidae|BOLD:AAD0784
Petroscirtes mitratus|LIFS878-08|Blenniidae|BOLD:AAE3283
Petroscirtes mitratus|LIFS877-08|Blenniidae|BOLD:AAE3283
Petroscirtes mitratus|LIFS875-08|Blenniidae|BOLD:AAE3283
Petroscirtes mitratus|LIFS876-08|Blenniidae|BOLD:AAE3283
Petroscirtes mitratus|LIFS722-08|Blenniidae|BOLD:AAE3283
Petroscirtes fallax|LIFS721-08|Blenniidae|BOLD:AAE6133
Petroscirtes fallax|LIFS723-08|Blenniidae|BOLD:AAE6133
Petroscirtes fallax|LIFS874-08|Blenniidae|BOLD:AAE6133
Petroscirtes fallax|LIFS720-08|Blenniidae|BOLD:AAE6133
Petroscirtes variabilis|LIFS715-08|Blenniidae|BOLD:ABZ5090
Petroscirtes variabilis|LIFS714-08|Blenniidae|BOLD:ABZ5090
Petroscirtes variabilis|LIFS711-08|Blenniidae|BOLD:ABZ5090
Petroscirtes variabilis|LIFS712-08|Blenniidae|BOLD:ABZ5090
Petroscirtes variabilis|LIFS713-08|Blenniidae|BOLD:ABZ5090
Diplogrammus goramensis|LIFSA147-08|Callionymidae|BOLD:AAI9957
Callionymidae|LIFS869-08|Callionymidae|BOLD:AAF9888
Sebastapistes strongia|LIFSA023-08|Scorpaenidae|BOLD:AAC4545
Sebastapistes strongia|LIFSA022-08|Scorpaenidae|BOLD:AAC4545
Sebastapistes strongia|LIFSA026-08|Scorpaenidae|BOLD:AAC4545
Sebastapistes strongia|LIFSA024-08|Scorpaenidae|BOLD:AAC4545
Sebastapistes strongia|LIFSA025-08|Scorpaenidae|BOLD:AAC4545
Sebastapistes strongia|LIFS522-08|Scorpaenidae|BOLD:AAC4545
Enneapterygius philippinus|LIFSA056-08|Tripterygiidae|BOLD:AAC0703
Enneapterygius philippinus|LIFSA017-08|Tripterygiidae|BOLD:AAC0703
Enneapterygius philippinus|LIFSA016-08|Tripterygiidae|BOLD:AAC0703
Enneapterygius philippinus|LIFSA014-08|Tripterygiidae|BOLD:AAC0703
Enneapterygius philippinus|LIFSA015-08|Tripterygiidae|BOLD:AAC0703
Enneapterygius philippinus|LIFSA027-08|Tripterygiidae|BOLD:AAC0703
Meiacanthus atrodorsalis|LIFS833-08|Blenniidae|BOLD:AAF3468
Meiacanthus atrodorsalis|LIFS823-08|Blenniidae|BOLD:AAF3468
Enneapterygius hemimelas|LIFSA155-08|Tripterygiidae|BOLD:AAC0704
Enneapterygius hemimelas|LIFSA154-08|Tripterygiidae|BOLD:AAC0704
Enneapterygius hemimelas|LIFSA153-08|Tripterygiidae|BOLD:AAC0704
Enneapterygius hemimelas|LIFSA149-08|Tripterygiidae|BOLD:AAC0704
Enneapterygius hemimelas|LIFSA067-08|Tripterygiidae|BOLD:AAC0704
Enneapterygius hemimelas|LIFSA066-08|Tripterygiidae|BOLD:AAC0704
Enneapterygius hemimelas|LIFSA064-08|Tripterygiidae|BOLD:AAC0704
Enneapterygius hemimelas|LIFSA150-08|Tripterygiidae|BOLD:AAC0704
Enneapterygius hemimelas|LIFSA156-08|Tripterygiidae|BOLD:AAC0704
Enneapterygius cf. hemimelas|LIFSA151-08|Tripterygiidae|BOLD:AAC0704
Enneapterygius hemimelas|LIFSA152-08|Tripterygiidae|BOLD:AAC0704
Ucla xenogrammus|LIFS554-08|Tripterygiidae|BOLD:AAH7696
Enneapterygius elegans|LIFSA013-08|Tripterygiidae|BOLD:AAC0702
Enneapterygius elegans|LIFS908-08|Tripterygiidae|BOLD:AAC0702
Atrosalarias fuscus|LIFSA055-08|Blenniidae|BOLD:AAC5976
Salarias guttatus|LIFSA062-08|Blenniidae|BOLD:AAB7192
Salarias alboguttatus|LIFS649-08|Blenniidae|BOLD:AAE7773
Salarias alboguttatus|LIFSA052-08|Blenniidae|BOLD:AAE7773
Salarias alboguttatus|LIFS967-08|Blenniidae|BOLD:AAE7773
Salarias fasciatus|LIFSA050-08|Blenniidae|BOLD:AAB7191
Salarias fasciatus|LIFSA051-08|Blenniidae|BOLD:AAB7191
Salarias fasciatus|LIFS979-08|Blenniidae|BOLD:AAB7191
Salarias fasciatus|LIFS980-08|Blenniidae|BOLD:AAB7191
Salarias cf. fasciatus|LIFS952-08|Blenniidae|BOLD:AAB7190
Balistoides viridescens|LIFSA068-08|Balistidae|BOLD:AAD0474
Balistoides viridescens|LIFS870-08|Balistidae|BOLD:AAD0474
Pseudobalistes fuscus|LIFS305-08|Balistidae|BOLD:ACZ6203
Abalistes stellatus|LIFS222-08|Balistidae|BOLD:ACK7828
Sufflamen chrysopterum|LIFS927-08|Balistidae|BOLD:AAB1339
Sufflamen chrysopterum|LIFS935-08|Balistidae|BOLD:AAB1339
Sufflamen chrysopterum|LIFS555-08|Balistidae|BOLD:AAB1339
Sufflamen chrysopterum|LIFSA262-08|Balistidae|BOLD:AAB1339
Sufflamen chrysopterum|LIFSA029-08|Balistidae|BOLD:AAB1339
Sufflamen chrysopterum|LIFS237-08|Balistidae|BOLD:AAB1339
Sufflamen chrysopterum|LIFS716-08|Balistidae|BOLD:AAB1339
Balistapus undulatus|LIFS601-08|Balistidae|BOLD:AAC2755
Rhinecanthus aculeatus|LIFSA035-08|Balistidae|BOLD:AAB6992
Rhinecanthus aculeatus|LIFS945-08|Balistidae|BOLD:AAB6992
Rhinecanthus aculeatus|LIFS415-08|Balistidae|BOLD:AAB6992
Gerres longirostris|LIFS413-08|Gerreidae|BOLD:AAE6360
Gerres erythrourus|LIFS702-08|Gerreidae|BOLD:AAF5833
Gerres erythrourus|LIFS701-08|Gerreidae|BOLD:AAF5833
Gerres oyena|LIFS692-08|Gerreidae|BOLD:AAC1289
Gerres oyena|LIFS690-08|Gerreidae|BOLD:AAC1289

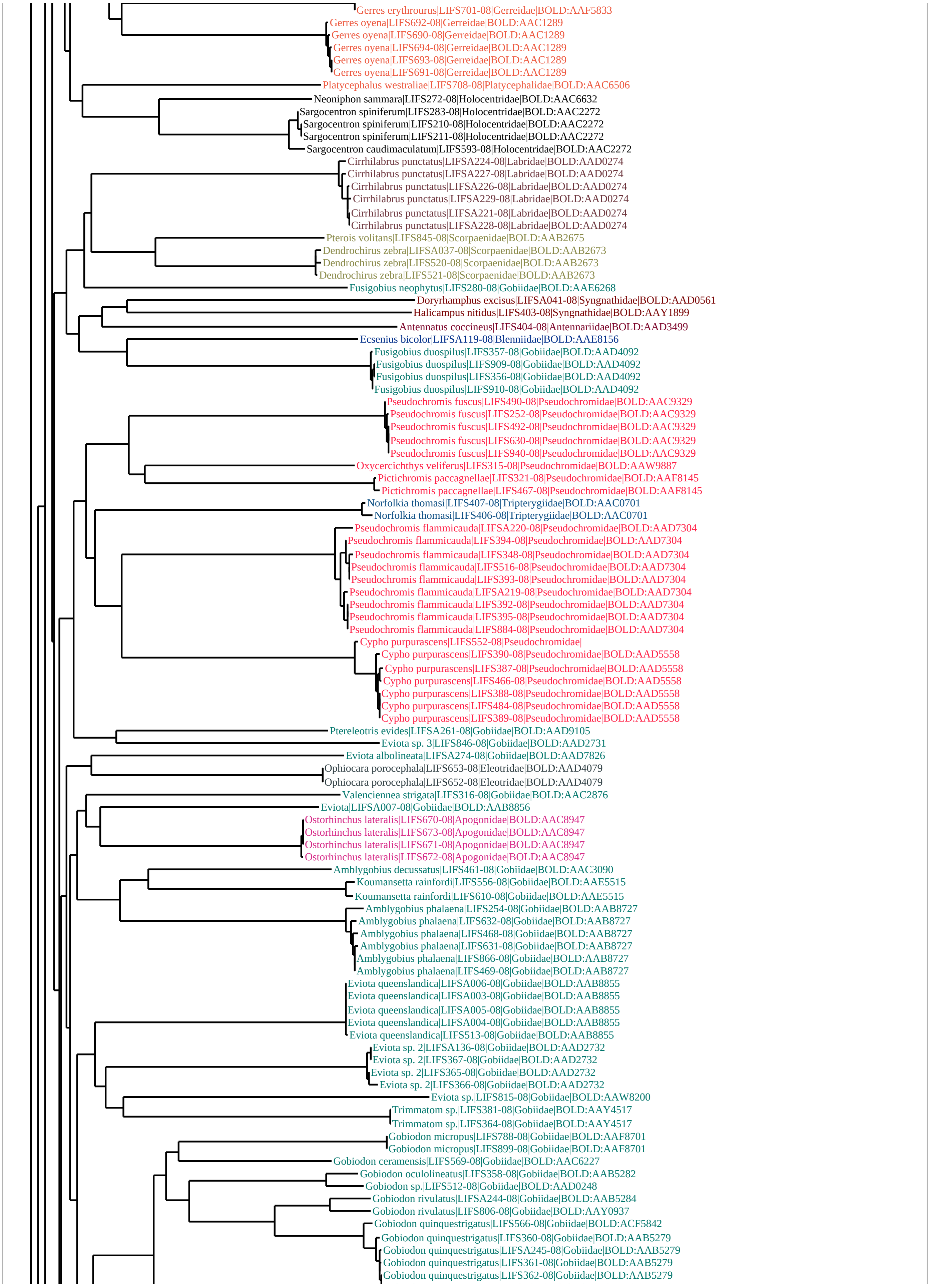
Gerres erythrourus|LIFS701-08|Gerreidae|BOLD:AAF5833
Gerres oyena|LIFS692-08|Gerreidae|BOLD:AAC1289
Gerres oyena|LIFS690-08|Gerreidae|BOLD:AAC1289
Gerres oyena|LIFS694-08|Gerreidae|BOLD:AAC1289
Gerres oyena|LIFS693-08|Gerreidae|BOLD:AAC1289
Gerres oyena|LIFS691-08|Gerreidae|BOLD:AAC1289
Platycephalus westraliae|LIFS708-08|Platycephalidae|BOLD:AAC6506
Neoniphon sammara|LIFS272-08|Holocentridae|BOLD:AAC6632
Sargocentron spiniferum|LIFS283-08|Holocentridae|BOLD:AAC2272
Sargocentron spiniferum|LIFS210-08|Holocentridae|BOLD:AAC2272
Sargocentron spiniferum|LIFS211-08|Holocentridae|BOLD:AAC2272
Sargocentron caudimaculatum|LIFS593-08|Holocentridae|BOLD:AAC2272
Cirrhilabrus punctatus|LIFSA224-08|Labridae|BOLD:AAD0274
Cirrhilabrus punctatus|LIFSA227-08|Labridae|BOLD:AAD0274
Cirrhilabrus punctatus|LIFSA226-08|Labridae|BOLD:AAD0274
Cirrhilabrus punctatus|LIFSA229-08|Labridae|BOLD:AAD0274
Cirrhilabrus punctatus|LIFSA221-08|Labridae|BOLD:AAD0274
Cirrhilabrus punctatus|LIFSA228-08|Labridae|BOLD:AAD0274
Pterois volitans|LIFS845-08|Scorpaenidae|BOLD:AAB2675
Dendrochirus zebra|LIFSA037-08|Scorpaenidae|BOLD:AAB2673
Dendrochirus zebra|LIFS520-08|Scorpaenidae|BOLD:AAB2673
Dendrochirus zebra|LIFS521-08|Scorpaenidae|BOLD:AAB2673
Fusigobius neophytus|LIFS280-08|Gobiidae|BOLD:AAE6268
Doryrhamphus excisus|LIFSA041-08|Syngnathidae|BOLD:AAD0561
Halicampus nitidus|LIFS403-08|Syngnathidae|BOLD:AAY1899
Antennatus coccineus|LIFS404-08|Antennariidae|BOLD:AAD3499
Ecsenius bicolor|LIFSA119-08|Blenniidae|BOLD:AAE8156
Fusigobius duospilus|LIFS357-08|Gobiidae|BOLD:AAD4092
Fusigobius duospilus|LIFS909-08|Gobiidae|BOLD:AAD4092
Fusigobius duospilus|LIFS356-08|Gobiidae|BOLD:AAD4092
Fusigobius duospilus|LIFS910-08|Gobiidae|BOLD:AAD4092
Pseudochromis fuscus|LIFS490-08|Pseudochromidae|BOLD:AAC9329
Pseudochromis fuscus|LIFS252-08|Pseudochromidae|BOLD:AAC9329
Pseudochromis fuscus|LIFS492-08|Pseudochromidae|BOLD:AAC9329
Pseudochromis fuscus|LIFS630-08|Pseudochromidae|BOLD:AAC9329
Pseudochromis fuscus|LIFS940-08|Pseudochromidae|BOLD:AAC9329
Oxycercichthys veliferus|LIFS315-08|Pseudochromidae|BOLD:AAW9887
Pictichromis paccagnellae|LIFS321-08|Pseudochromidae|BOLD:AAF8145
Pictichromis paccagnellae|LIFS467-08|Pseudochromidae|BOLD:AAF8145
Norfolkia thomasi|LIFS407-08|Tripterygiidae|BOLD:AAC0701
Norfolkia thomasi|LIFS406-08|Tripterygiidae|BOLD:AAC0701
Pseudochromis flammicauda|LIFSA220-08|Pseudochromidae|BOLD:AAD7304
Pseudochromis flammicauda|LIFS394-08|Pseudochromidae|BOLD:AAD7304
Pseudochromis flammicauda|LIFS348-08|Pseudochromidae|BOLD:AAD7304
Pseudochromis flammicauda|LIFS516-08|Pseudochromidae|BOLD:AAD7304
Pseudochromis flammicauda|LIFS393-08|Pseudochromidae|BOLD:AAD7304
Pseudochromis flammicauda|LIFSA219-08|Pseudochromidae|BOLD:AAD7304
Pseudochromis flammicauda|LIFS392-08|Pseudochromidae|BOLD:AAD7304
Pseudochromis flammicauda|LIFS395-08|Pseudochromidae|BOLD:AAD7304
Pseudochromis flammicauda|LIFS884-08|Pseudochromidae|BOLD:AAD7304
Cypho purpurascens|LIFS552-08|Pseudochromidae|
Cypho purpurascens|LIFS390-08|Pseudochromidae|BOLD:AAD5558
Cypho purpurascens|LIFS387-08|Pseudochromidae|BOLD:AAD5558
Cypho purpurascens|LIFS466-08|Pseudochromidae|BOLD:AAD5558
Cypho purpurascens|LIFS388-08|Pseudochromidae|BOLD:AAD5558
Cypho purpurascens|LIFS484-08|Pseudochromidae|BOLD:AAD5558
Cypho purpurascens|LIFS389-08|Pseudochromidae|BOLD:AAD5558
Ptereleotris evides|LIFSA261-08|Gobiidae|BOLD:AAD9105
Eviota sp. 3|LIFS846-08|Gobiidae|BOLD:AAD2731
Eviota albolineata|LIFSA274-08|Gobiidae|BOLD:AAD7826
Ophiocara porocephala|LIFS653-08|Eleotridae|BOLD:AAD4079
Ophiocara porocephala|LIFS652-08|Eleotridae|BOLD:AAD4079
Valenciennea strigata|LIFS316-08|Gobiidae|BOLD:AAC2876
Eviota|LIFSA007-08|Gobiidae|BOLD:AAB8856
Ostorhinchus lateralis|LIFS670-08|Apogonidae|BOLD:AAC8947
Ostorhinchus lateralis|LIFS673-08|Apogonidae|BOLD:AAC8947
Ostorhinchus lateralis|LIFS671-08|Apogonidae|BOLD:AAC8947
Ostorhinchus lateralis|LIFS672-08|Apogonidae|BOLD:AAC8947
Amblygobius decussatus|LIFS461-08|Gobiidae|BOLD:AAC3090
Koumansetta rainfordi|LIFS556-08|Gobiidae|BOLD:AAE5515
Koumansetta rainfordi|LIFS610-08|Gobiidae|BOLD:AAE5515
Amblygobius phalaena|LIFS254-08|Gobiidae|BOLD:AAB8727
Amblygobius phalaena|LIFS632-08|Gobiidae|BOLD:AAB8727
Amblygobius phalaena|LIFS468-08|Gobiidae|BOLD:AAB8727
Amblygobius phalaena|LIFS631-08|Gobiidae|BOLD:AAB8727
Amblygobius phalaena|LIFS866-08|Gobiidae|BOLD:AAB8727
Amblygobius phalaena|LIFS469-08|Gobiidae|BOLD:AAB8727
Eviota queenslandica|LIFSA006-08|Gobiidae|BOLD:AAB8855
Eviota queenslandica|LIFSA003-08|Gobiidae|BOLD:AAB8855
Eviota queenslandica|LIFSA005-08|Gobiidae|BOLD:AAB8855
Eviota queenslandica|LIFSA004-08|Gobiidae|BOLD:AAB8855
Eviota queenslandica|LIFS513-08|Gobiidae|BOLD:AAB8855
Eviota sp. 2|LIFSA136-08|Gobiidae|BOLD:AAD2732
Eviota sp. 2|LIFS367-08|Gobiidae|BOLD:AAD2732
Eviota sp. 2|LIFS365-08|Gobiidae|BOLD:AAD2732
Eviota sp. 2|LIFS366-08|Gobiidae|BOLD:AAD2732
Eviota sp.|LIFS815-08|Gobiidae|BOLD:AAW8200
Trimmatom sp.|LIFS381-08|Gobiidae|BOLD:AAY4517
Trimmatom sp.|LIFS364-08|Gobiidae|BOLD:AAY4517
Gobiodon micropus|LIFS788-08|Gobiidae|BOLD:AAF8701
Gobiodon micropus|LIFS899-08|Gobiidae|BOLD:AAF8701
Gobiodon ceramensis|LIFS569-08|Gobiidae|BOLD:AAC6227
Gobiodon oculolineatus|LIFS358-08|Gobiidae|BOLD:AAB5282
Gobiodon sp.|LIFS512-08|Gobiidae|BOLD:AAD0248
Gobiodon rivulatus|LIFSA244-08|Gobiidae|BOLD:AAB5284
Gobiodon rivulatus|LIFS806-08|Gobiidae|BOLD:AAY0937
Gobiodon quinquestrigatus|LIFS566-08|Gobiidae|BOLD:ACF5842
Gobiodon quinquestrigatus|LIFS360-08|Gobiidae|BOLD:AAB5279
Gobiodon quinquestrigatus|LIFSA245-08|Gobiidae|BOLD:AAB5279
Gobiodon quinquestrigatus|LIFS361-08|Gobiidae|BOLD:AAB5279
Gobiodon quinquestrigatus|LIFS362-08|Gobiidae|BOLD:AAB5279

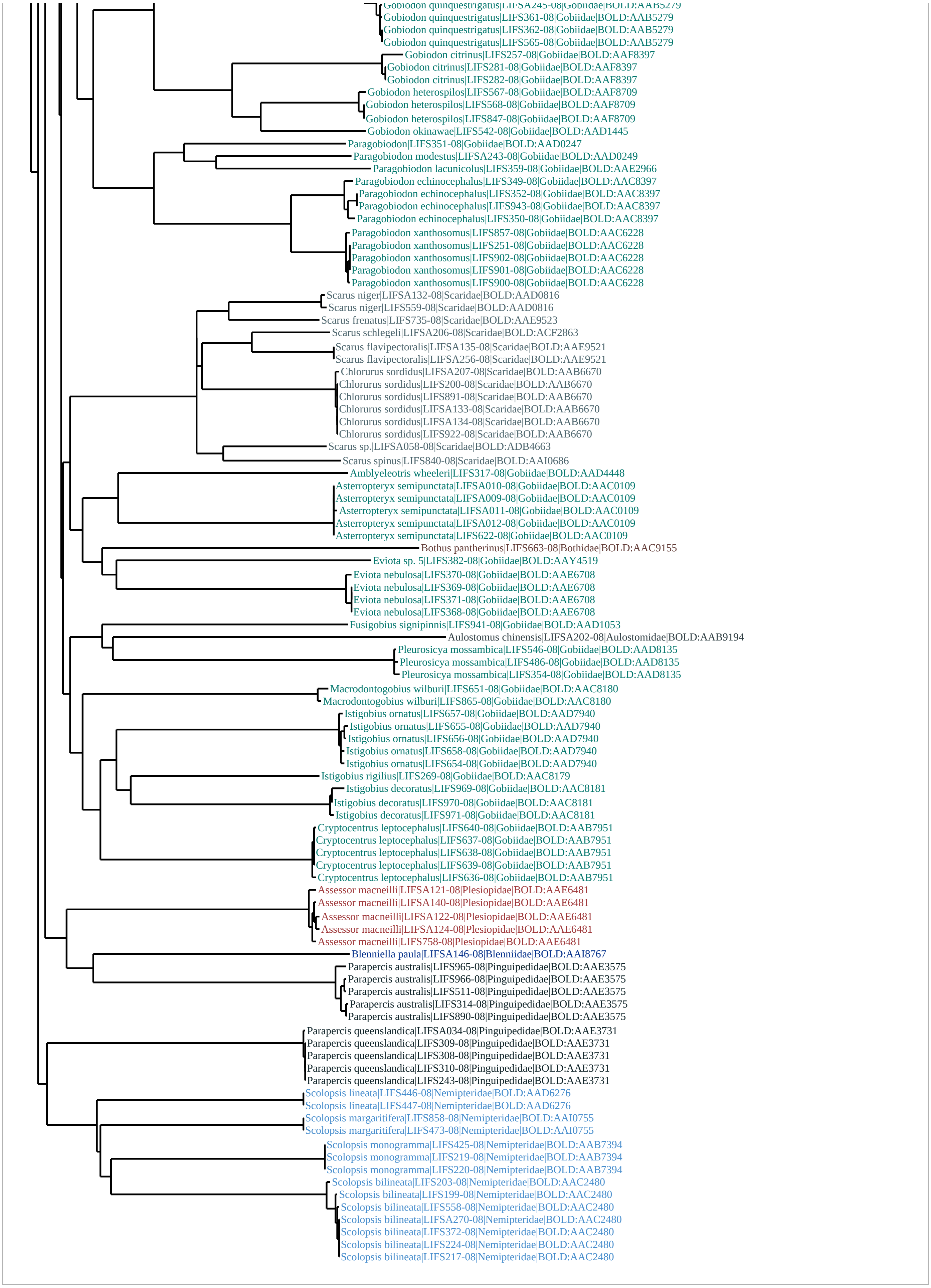
Gobiodon quinquestrigatus|LIFSA245-08|Gobiidae|BOLD:AAB5279
Gobiodon quinquestrigatus|LIFS361-08|Gobiidae|BOLD:AAB5279
Gobiodon quinquestrigatus|LIFS362-08|Gobiidae|BOLD:AAB5279
Gobiodon quinquestrigatus|LIFS565-08|Gobiidae|BOLD:AAB5279
Gobiodon citrinus|LIFS257-08|Gobiidae|BOLD:AAF8397
Gobiodon citrinus|LIFS281-08|Gobiidae|BOLD:AAF8397
Gobiodon citrinus|LIFS282-08|Gobiidae|BOLD:AAF8397
Gobiodon heterospilos|LIFS567-08|Gobiidae|BOLD:AAF8709
Gobiodon heterospilos|LIFS568-08|Gobiidae|BOLD:AAF8709
Gobiodon heterospilos|LIFS847-08|Gobiidae|BOLD:AAF8709
Gobiodon okinawae|LIFS542-08|Gobiidae|BOLD:AAD1445
Paragobiodon|LIFS351-08|Gobiidae|BOLD:AAD0247
Paragobiodon modestus|LIFSA243-08|Gobiidae|BOLD:AAD0249
Paragobiodon lacunicolus|LIFS359-08|Gobiidae|BOLD:AAE2966
Paragobiodon echinocephalus|LIFS349-08|Gobiidae|BOLD:AAC8397
Paragobiodon echinocephalus|LIFS352-08|Gobiidae|BOLD:AAC8397
Paragobiodon echinocephalus|LIFS943-08|Gobiidae|BOLD:AAC8397
Paragobiodon echinocephalus|LIFS350-08|Gobiidae|BOLD:AAC8397
Paragobiodon xanthosomus|LIFS857-08|Gobiidae|BOLD:AAC6228
Paragobiodon xanthosomus|LIFS251-08|Gobiidae|BOLD:AAC6228
Paragobiodon xanthosomus|LIFS902-08|Gobiidae|BOLD:AAC6228
Paragobiodon xanthosomus|LIFS901-08|Gobiidae|BOLD:AAC6228
Paragobiodon xanthosomus|LIFS900-08|Gobiidae|BOLD:AAC6228
Scarus niger|LIFSA132-08|Scaridae|BOLD:AAD0816
Scarus niger|LIFS559-08|Scaridae|BOLD:AAD0816
Scarus frenatus|LIFS735-08|Scaridae|BOLD:AAE9523
Scarus schlegeli|LIFSA206-08|Scaridae|BOLD:ACF2863
Scarus flavipectoralis|LIFSA135-08|Scaridae|BOLD:AAE9521
Scarus flavipectoralis|LIFSA256-08|Scaridae|BOLD:AAE9521
Chlorurus sordidus|LIFSA207-08|Scaridae|BOLD:AAB6670
Chlorurus sordidus|LIFS200-08|Scaridae|BOLD:AAB6670
Chlorurus sordidus|LIFS891-08|Scaridae|BOLD:AAB6670
Chlorurus sordidus|LIFSA133-08|Scaridae|BOLD:AAB6670
Chlorurus sordidus|LIFSA134-08|Scaridae|BOLD:AAB6670
Chlorurus sordidus|LIFS922-08|Scaridae|BOLD:AAB6670
Scarus sp.|LIFSA058-08|Scaridae|BOLD:ADB4663
Scarus spinus|LIFS840-08|Scaridae|BOLD:AAI0686
Amblyeleotris wheeleri|LIFS317-08|Gobiidae|BOLD:AAD4448
Asterropteryx semipunctata|LIFSA010-08|Gobiidae|BOLD:AAC0109
Asterropteryx semipunctata|LIFSA009-08|Gobiidae|BOLD:AAC0109
Asterropteryx semipunctata|LIFSA011-08|Gobiidae|BOLD:AAC0109
Asterropteryx semipunctata|LIFSA012-08|Gobiidae|BOLD:AAC0109
Asterropteryx semipunctata|LIFS622-08|Gobiidae|BOLD:AAC0109
Bothus pantherinus|LIFS663-08|Bothidae|BOLD:AAC9155
Eviota sp. 5|LIFS382-08|Gobiidae|BOLD:AAY4519
Eviota nebulosa|LIFS370-08|Gobiidae|BOLD:AAE6708
Eviota nebulosa|LIFS369-08|Gobiidae|BOLD:AAE6708
Eviota nebulosa|LIFS371-08|Gobiidae|BOLD:AAE6708
Eviota nebulosa|LIFS368-08|Gobiidae|BOLD:AAE6708
Fusigobius signipinnis|LIFS941-08|Gobiidae|BOLD:AAD1053
Aulostomus chinensis|LIFSA202-08|Aulostomidae|BOLD:AAB9194
Pleurosicya mossambica|LIFS546-08|Gobiidae|BOLD:AAD8135
Pleurosicya mossambica|LIFS486-08|Gobiidae|BOLD:AAD8135
Pleurosicya mossambica|LIFS354-08|Gobiidae|BOLD:AAD8135
Macrodontogobius wilburi|LIFS651-08|Gobiidae|BOLD:AAC8180
Macrodontogobius wilburi|LIFS865-08|Gobiidae|BOLD:AAC8180
Istigobius ornatus|LIFS657-08|Gobiidae|BOLD:AAD7940
Istigobius ornatus|LIFS655-08|Gobiidae|BOLD:AAD7940
Istigobius ornatus|LIFS656-08|Gobiidae|BOLD:AAD7940
Istigobius ornatus|LIFS658-08|Gobiidae|BOLD:AAD7940
Istigobius ornatus|LIFS654-08|Gobiidae|BOLD:AAD7940
Istigobius rigilius|LIFS269-08|Gobiidae|BOLD:AAC8179
Istigobius decoratus|LIFS969-08|Gobiidae|BOLD:AAC8181
Istigobius decoratus|LIFS970-08|Gobiidae|BOLD:AAC8181
Istigobius decoratus|LIFS971-08|Gobiidae|BOLD:AAC8181
Cryptocentrus leptocephalus|LIFS640-08|Gobiidae|BOLD:AAB7951
Cryptocentrus leptocephalus|LIFS637-08|Gobiidae|BOLD:AAB7951
Cryptocentrus leptocephalus|LIFS638-08|Gobiidae|BOLD:AAB7951
Cryptocentrus leptocephalus|LIFS639-08|Gobiidae|BOLD:AAB7951
Cryptocentrus leptocephalus|LIFS636-08|Gobiidae|BOLD:AAB7951
Assessor macneilli|LIFSA121-08|Plesiopidae|BOLD:AAE6481
Assessor macneilli|LIFSA140-08|Plesiopidae|BOLD:AAE6481
Assessor macneilli|LIFSA122-08|Plesiopidae|BOLD:AAE6481
Assessor macneilli|LIFSA124-08|Plesiopidae|BOLD:AAE6481
Assessor macneilli|LIFS758-08|Plesiopidae|BOLD:AAE6481
Blenniella paula|LIFSA146-08|Blenniidae|BOLD:AAI8767
Parapercis australis|LIFS965-08|Pinguipedidae|BOLD:AAE3575
Parapercis australis|LIFS966-08|Pinguipedidae|BOLD:AAE3575
Parapercis australis|LIFS511-08|Pinguipedidae|BOLD:AAE3575
Parapercis australis|LIFS314-08|Pinguipedidae|BOLD:AAE3575
Parapercis australis|LIFS890-08|Pinguipedidae|BOLD:AAE3575
Parapercis queenslandica|LIFSA034-08|Pinguipedidae|BOLD:AAE3731
Parapercis queenslandica|LIFS309-08|Pinguipedidae|BOLD:AAE3731
Parapercis queenslandica|LIFS308-08|Pinguipedidae|BOLD:AAE3731
Parapercis queenslandica|LIFS310-08|Pinguipedidae|BOLD:AAE3731
Parapercis queenslandica|LIFS243-08|Pinguipedidae|BOLD:AAE3731
Scolopsis lineata|LIFS446-08|Nemipteridae|BOLD:AAD6276
Scolopsis lineata|LIFS447-08|Nemipteridae|BOLD:AAD6276
Scolopsis margaritifera|LIFS858-08|Nemipteridae|BOLD:AAI0755
Scolopsis margaritifera|LIFS473-08|Nemipteridae|BOLD:AAI0755
Scolopsis monogramma|LIFS425-08|Nemipteridae|BOLD:AAB7394
Scolopsis monogramma|LIFS219-08|Nemipteridae|BOLD:AAB7394
Scolopsis monogramma|LIFS220-08|Nemipteridae|BOLD:AAB7394
Scolopsis bilineata|LIFS203-08|Nemipteridae|BOLD:AAC2480
Scolopsis bilineata|LIFS199-08|Nemipteridae|BOLD:AAC2480
Scolopsis bilineata|LIFS558-08|Nemipteridae|BOLD:AAC2480
Scolopsis bilineata|LIFSA270-08|Nemipteridae|BOLD:AAC2480
Scolopsis bilineata|LIFS372-08|Nemipteridae|BOLD:AAC2480
Scolopsis bilineata|LIFS224-08|Nemipteridae|BOLD:AAC2480
Scolopsis bilineata|LIFS217-08|Nemipteridae|BOLD:AAC2480
